# Precision Fc remodeling via glycosylation-competent CHO display enables ultra-selective FcγRIIIa targeting and enhanced antitumor activity

**DOI:** 10.1186/s13036-026-00671-8

**Published:** 2026-04-01

**Authors:** Migyeong Jo, Suyeon Kim, Sanghwan Ko, Munsu Kyung, Seunghyeon Lee, Woo Hyung Ko, Wonju Lee, Sang Taek Jung

**Affiliations:** 1 Department of Chemical and Biological Engineering, College of Engineering, Seoul National University, Seoul, 08826 Republic of Korea; 2 Institute of Chemical Processes, Seoul National University, Seoul, 08826 Republic of Korea; 3 Department of Biomedical Sciences, Graduate School, Korea University, Seoul, 02841 Republic of Korea; 4 Interdisciplinary Program for Bioengineering, Seoul National University, Seoul, 08826 Republic of Korea; 5 BioMAX, Seoul National University, Seoul, 08826 Republic of Korea; 6 Seoul National University Medical Research Center (SNUMRC), Seoul, 03080 Republic of Korea; 7 Present Address: Department of Chemical Engineering, University of Texas at Austin, Austin, TX 78712 USA; 8 Present Address: Manufacturing Science & Technology Team, Manufacturing Science Group2, Samsung Bioepis, Incheon, Republic of Korea

**Keywords:** Therapeutic IgG antibody, FcγRIIIa, CHO-based mammalian display, Fc engineering, Target cell lysis

## Abstract

**Background:**

Improving tumor cell clearance by therapeutic antibodies remains a translational bottleneck because wild-type IgG1 Fc typically elicits suboptimal NK cell–mediated antibody-dependent cellular cytotoxicity (ADCC), necessitating Fc engineering to enhance activating FcγR engagement while preserving antigen specificity and manufacturability. FcγRIIIa (CD16A) is the principal activating receptor on NK cells, and its clinical relevance is underscored by the FCGR3A-158V/F polymorphism, which modulates IgG1 Fc affinity and therapeutic response. Because FcγRIIIa engagement critically depends on Fc glycosylation, microbial display platforms lacking mammalian glycan processing are limited in capturing Fc–FcγRIIIa energetics and selectivity.

**Results:**

We developed a glycosylation-integrated Fc engineering platform using CHO surface display to screen glycosylated Fc libraries in a post-translationally accurate context. Multiparameter flow-cytometric selection with FcγRIIIa binding and FcγRIIb counter-screening enabled iterative isolation of PS-series Fc variants with ultra-selective, allotype-compatible FcγRIIIa recognition. Lead variants achieved 262-fold (158V) and 497-fold (158F) FcγRIIIa affinity gains while reducing FcγRIIb binding by up to 4.2-fold, expanding activating-to-inhibitory selectivity up to 2,096. This exceeded clinically deployed FcγRIIIa-enhancing Fc variants by 525-fold and 108-fold, respectively, in activating-to-inhibitory selectivity, compared with DE (S239D/I332E; used in tafasitamab) and VLPLL (L235V/F243L/R292P/Y300L/P396L; used in margetuximab). When grafted onto trastuzumab, PS variants enhanced NK cytotoxicity and improved tumor control in a trastuzumab-refractory xenograft model. Modular transfer to cetuximab and rituximab also increased cytotoxic activity.

**Conclusions:**

This study establishes a glycosylation-integrated CHO display platform for precision FcγRIIIa-targeted Fc engineering, generating modular effector domains with broad allotype compatibility, minimal FcγRIIb binding, and robust therapeutic potential in cancer immunotherapy.

**Supplementary Information:**

The online version contains supplementary material available at 10.1186/s13036-026-00671-8.

## Background

Monoclonal antibodies (mAbs) have revolutionized immunotherapy by enabling precise targeting, favorable safety profiles, and extended serum half-life. As of 2024, over 100 mAbs have been approved by the US Food and Drug Administration (FDA), with their use in both clinical and commercial settings continuing to expand, underscoring the escalating demand for next-generation antibody therapeutics [1]. The clinical efficacy of IgG antibodies is largely mediated through their Fc region, which engages Fc gamma receptors (FcγRs) expressed across key immune effector cells, including NK cells, macrophages, neutrophils, and dendritic cells [2, 3]. Human FcγRs include FcγRI (CD64), FcγRIIa (CD32a), FcγRIIb (CD32b), FcγRIIIa (CD16A), and FcγRIIIb (CD16B), each characterized by distinct expression profiles and immunomodulatory functions [4–6]. Among them, FcγRIIIa (CD16A) is the principal activating receptor for NK cells, governing antibody-dependent cellular cytotoxicity (ADCC) [7] via ITAM-bearing adaptor molecules such as CD3ζ or FcεRIγ [8–10]. Unlike other NK receptors that recognize pathogen-associated or stress-induced ligands, FcγRIIIa uniquely empowers NK cells to detect and eliminate IgG-opsonized tumor cells, representing a specialized evolutionary adaptation for antitumor immunity [11, 12].

The therapeutic significance of Fc–FcγRIIIa affinity is exemplified by the FcγRIIIa-158V/F polymorphism. The 158V allotype confers higher affinity for IgG1 Fc than 158F variant [5], and correlates with improved clinical responses to trastuzumab, rituximab, and cetuximab [13–15]. These data validate FcγRIIIa affinity as a predictive determinant of antibody efficacy, particularly in ADCC-dependent indications.

To enhance FcγRIIIa engagement, glycoengineering and protein engineering strategies have been pursued. Glycoengineering primarily targets the conserved Asn297 glycan, where defucosylation markedly increases FcγRIIIa binding [16, 17]. However, glycan profiles are highly sensitive to host cell lineage and bioprocess conditions, posing challenges for batch-to-batch consistency [18, 19]. Alternatively, protein engineering has yielded Fc variants with enhanced FcγRIIIa affinity through targeted substitutions, as exemplified by DE (S239D/I332E) and VLPLL (L235V/F243L/R292P/Y300L/P396L) variants incorporated into FDA-approved antibodies: tafasitamab (Mojuvi^®^, 2020) [20] and margetuximab (Margenza^®^, 2020) [21]. However, despite these advances, the discovery platforms commonly employed—such as phage and bacterial display—lack glycosylation machinery altogether [22], while yeast display yields high-mannose glycans that deviate from human patterns [23], thereby limiting their post-translational fidelity in faithfully recapitulating FcγRIIIa interactions.

To address these limitations, we engineered a CHO-based mammalian surface display platform that enables Fc screening under glycosylation-competent, post-translationally accurate conditions. By anchoring Fc libraries to the cell surface via PDGFR transmembrane domains, this system supports flow cytometry–based multiparameter selection, integrating positive selection for FcγRIIIa binding with counter-selection against FcγRIIb. This architecture enables directed evolution of Fc domains within a mammalian expression context, coupling combinatorial sequence diversification with native glycan processing to optimize effector receptor engagement. Using this directed evolution framework, we identified novel glycosylated Fc variants exhibiting exceptional FcγRIIIa affinity alongside diminished FcγRIIb interaction. Incorporation into trastuzumab yielded variants with superior ADCC activity in vitro and enhanced antitumor efficacy in a trastuzumab-resistant xenograft model.

These findings establish a glycosylation-competent Fc engineering strategy for precisely modulating FcγRIIIa binding, offering a modular engineering framework to generate next-generation antibody therapeutics with enhanced effector potency and broad therapeutic applicability.

## Methods

### Generation of a stably expressing CHO cell library of Fc variants via Flp recombination

Ten amino acid residues within the Fc region (position 239, 243, 247, 292, 300, 305, 330, 332, 339, and 396) were diversified using wild-type residues or previously reported mutations. The Fc gene was segmented into four fragments and amplified using the following primer sets: MJ#273/MJ#418–421, MJ#422/MJ#423–424, MJ#425–432/MJ#433–434, and MJ#435/MJ#274. These fragments were assembled into full-length Fc using overlap extension PCR with primers MJ#273/MJ#274 and Vent DNA polymerase, followed by *Sfi*I digestion. The resulting product was cloned into the *Sfi*I-digested pcDNA5-Igκ-Fc-FLAG-PDGFR vector and transformed into *Escherichia coli* Jude1 to generate the Fc variant library plasmid. The plasmids were co-transfected with the pOG44-Flp into Flp-in CHO cells™ (Thermo Fisher Scientific, Waltham, MA, USA) at a 1:9 ratio to mediate site-specific integration via FRT recombination. Stable integrants were selected using hygromycin B (500 µg/ml) in Ham’s F12 Nutrient Mix, GlutaMAX™ medium supplemented with 10% of FBS. This process generated a CHO cell library stably expressing membrane-anchored Fc variants.

### Biolayer interferometry (BLI) analysis for quantitation of trastuzumab-Fc variants

The Octet R8 instrument (Sartorius, Göttingen, Germany) was used to measure the equilibrium binding constants of trastuzumab-Fc variants. FAB2G biosensors were hydrated in distilled water for 10 min, followed by incubation with 500 nM trastuzumab-Fc variants for 300 s to allow antibody binding, after which the baseline was measured in PBS for 60 s. For the association phase, monomeric FcγRIIIa-158V-His, monomeric FcγRIIIa-158F-His, or monomeric FcγRIIb-His diluted in PBS was introduced to the captured trastuzumab-Fc variants and incubated for 30 s, followed by dissociation in PBS for either 30–300 s depending on steady-state or full kinetic analysis.

### Real-time ADCC assay for trastuzumab-Fc variants and cetuximab-Fc variants

The cytotoxic activity of trastuzumab and cetuximab Fc variants was assessed in real-time using the xCELLigence RTCA SP system (Agilent, Santa Clara, CA, USA). SK-BR-3 and MDA-MB-453 cells were used as HER2^+^ targets for trastuzumab; A431 cells were used as EGFR^+^ targets for cetuximab. Target cells (1 × 10^4^ SK-BR-3/MDA-MB-453 cells or 2 × 10^4^ A431) were seeded into E-plates (Agilent, Santa Clara, CA, USA) and cultured in RPMI 1640 medium with 10% FBS for 24 h. Freshly prepared peripheral blood mononuclear cells (PBMCs) were added together with Fc variant antibodies to engage target cells. Wells containing 2% Triton X-100, 1% SDS, 100 mM NaCl, and 1 mM EDTA served as positive lysis controls. Cytotoxicity was monitored by impedance-based cell index measurements and converted to percent lysis.

### ADCC assay using GFP-CD16-V/V-NK92 cells for rituximab-Fc variants

A total of 2 × 10⁴ Ramos cells and 4 × 10⁴ GFP-CD16-V/V-NK92 cells were co-incubated in a 96-well V-bottom plate with 50 pM rituximab-Fc variants for 4 h at an effector-to-target (E:T) ratio of 2:1. Ramos cells were cultured in RPMI 1640 medium supplemented with 10% FBS, and NK92 cells were maintained in MyeloCult™ H5100 medium (STEMCELL, Vancouver, British Columbia, Canada) supplemented with 100 U/ml of recombinant human IL-2 (Miltenyi Biotec, Bergisch Gladbach, Germany) and horse serum (Gibco, New Zealand origin). Following incubation, cells were stained with 5 nM SYTOX Red (Invitrogen, Waltham, MA, USA) to label dead cells. Samples were analyzed using a BD FACSLyric™ flow cytometer, and cytotoxicity was calculated as the percentage of dead tumor cells relative to the total tumor cell population.

### Live-cell imaging for analysis of cytotoxicity

To monitor ADCC in real-time, SK-BR-3 cells were washed with 1× PBS and resuspended in 2 µM CellTracker™ Red CMTPX dye (Thermo Fisher Scientific), followed by incubation at 37°C for 30 min to allow fluorescence labeling. After incubation, cells were washed with 1× PBS, resuspended in complete medium (phenol red-free RPMI supplemented with 10% fetal bovine serum and 1× Antibiotic-Antimycotic), and seeded at a density of 5,000 cells per well in black-walled, clear-bottom 96-well plate. Cells were then incubated for 16 h at 37°C in a 5% CO₂ atmosphere. PBMCs were isolated from healthy donor blood and stained with 5 µg/ml Hoechst 33342 for nuclear labeling and then added to target cells at an E:T ratio of 10:1 (50,000 PBMCs per well). Subsequently, antibodies were added to the wells at a final concentration of 5 µg/ml to induce ADCC. Caspase-3/7 Green Detection Reagent (2 µM) was added to monitor apoptosis. Real-time imaging was performed using a Lionheart FX Automated Live Cell Imager (BioTek). Fluorescence images were acquired every 60 min under 37°C and 5% CO₂, and image analysis was conducted using Gen5 software (BioTek).

### Analysis of in vivo efficacy in mouse xenograft

Five-week-old female BALB/c nude (CAnN.Cg-Foxn1 nu/CrlOri) mice (Orient Bio, Seongnam, Republic of Korea) were randomly assigned into groups (*n* = 10). JIMT-1 cells (5 × 10⁶ in 100 µl Opti-MEM) were subcutaneously implanted into the flank of each mouse. When tumors reached approximately 80 mm³, mice were treated intraperitoneally with Fc variant-bearing trastuzumab (10 mg/kg) twice weekly for six doses. Tumor volumes were measured three times weekly using electronic calipers and calculated as (length × width²)/2. Body weights were also recorded to monitor systemic toxicity. Mice were euthanized on day 46 post-implantation, and tumors were harvested and weighed. Group comparisons of tumor volume were evaluated using one-way ANOVA.

## Results

### Development of a CHO cell surface display platform for glycosylated Fc engineering

To improve the therapeutic efficacy of antibodies through enhanced antibody-dependent cellular cytotoxicity (ADCC), we investigated whether combining previously reported ADCC-enhancing Fc mutations could yield additive or synergistic effects. Specifically, we focused on three representative variants identified by independent platforms: S239D/A330L/I332E (Xencor, X) [24, 25], F243L/R292P/Y300L/V305I/P396L (MacroGenics, M_a_) [26], and P247I/A339Q (Mentrik Biotech, M_t_) [27]. We generated trastuzumab variants incorporating pairwise or triple combinations of these modules—including XM_a_, XM_t_, M_a_M_t_, and XM_a_M_t_—and assessed their binding profiles to hFcγRIIIa and hFcγRIIb (Supplementary Fig. 1). Unexpectedly, all combinatorial variants exhibited increased binding to the inhibitory receptor hFcγRIIb, which shares an overlapping epitope with hFcγRIIIa. Given that hFcγRIIb attenuates immune activation and competes with activating hFcγRs for antibody binding, its enhanced engagement likely compromises ADCC potency [28]. These results underscore the limitations of rational stacking of beneficial mutations and highlight the need for a screening platform that can assess a vast number of combinatorial Fc variant performances under glycosylation-dependent and receptor-specific conditions.

**Fig. 1:**
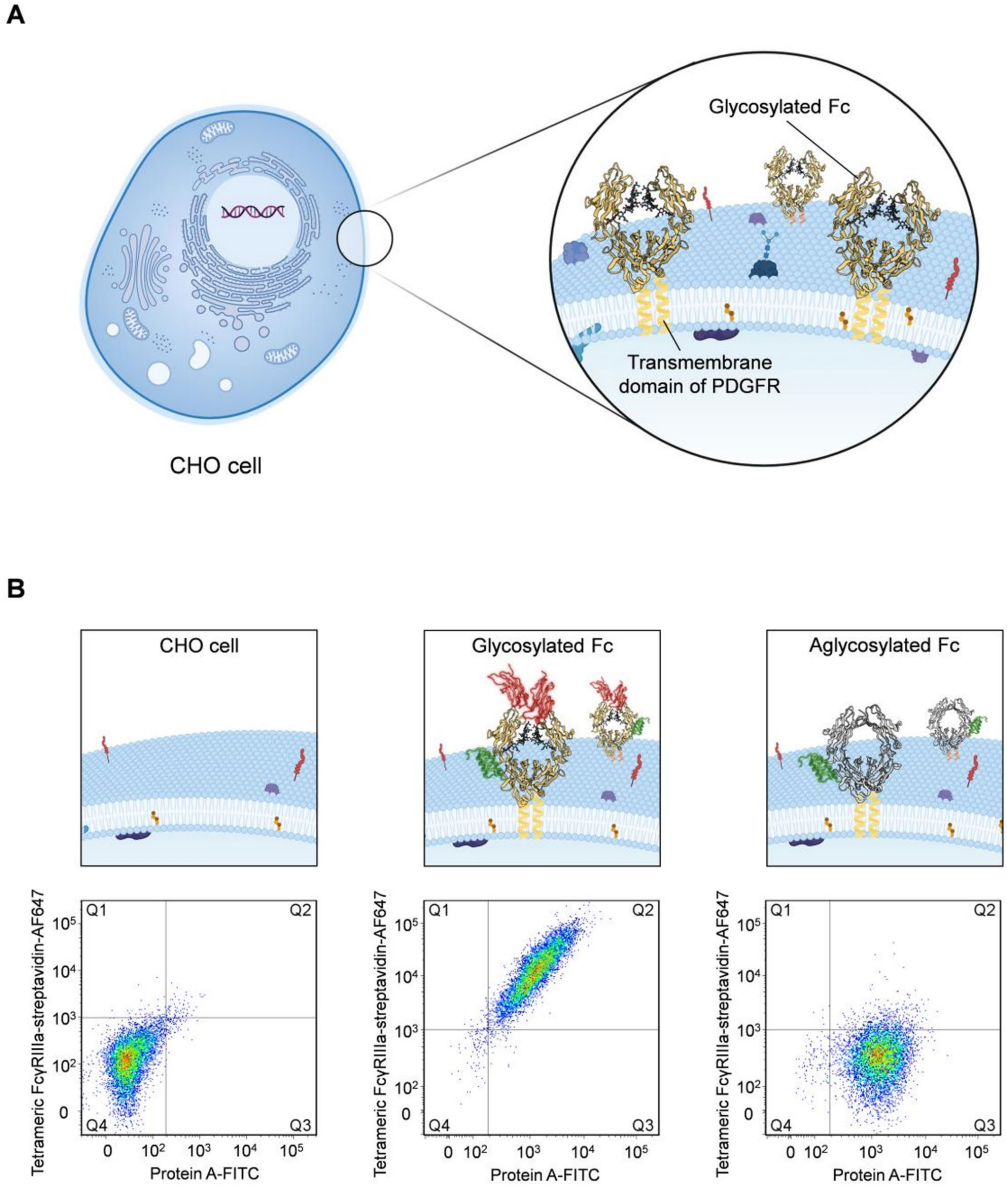
Schematic overview and validation of the CHO cell surface display system. (**A**) Schematic diagram illustrating the CHO cell surface display system established through FLP–FRT recombination. (**B**) Flow cytometry density plots demonstrating the successful establishment of stable CHO cell lines displaying either aglycosylated Fc (Fc-TL) or glycosylated Fc, as confirmed by functional binding to tetrameric FcγRIIIa-158V-streptavidin-AF647

To meet this need, we established a mammalian CHO cell-based display platform that enables high-throughput functional screening of glycosylated Fc variants under human-like glycosylation conditions. Using Flp recombinase–mediated site-specific recombination at a flippase recognition target (FRT) site, we generated stable CHO cell lines expressing membrane-anchored Fc variants fused to the PDGFR transmembrane domain (Fig. 1A and Supplementary Fig. 2). The constructs included wild-type glycosylated Fc a T299L mutant that disrupts the canonical *N*-linked glycosylation motif, yielding an aglycosylated Fc with diminished FcγR binding. Following co-transfection with the Flp recombinase–expressing plasmid and antibiotic selection, surface display of Fc was validated via flow cytometry using Protein A-FITC and hFcγRIIIa–streptavidin–Alexa Fluor 647 (AF647) (Fig. 1B, left). As expected, glycosylated Fc-expressing CHO cells bound both probes (Fig. 1B, middle), while aglycosylated Fc-expressing cells displayed strong Protein A–FITC signal but minimal hFcγRIIIa engagement (Fig. 1B, right). These results validate a glycosylation-competent CHO surface display platform suitable for high-throughput discovery of Fc variants with optimized hFcγRIIIa engagement and reduced hFcγRIIb binding. Importantly, this platform enables functional screening in a physiologically relevant context that closely mimics native human glycosylation. While our study focused on enhancing ADCC, this platform can be broadly applied to discover Fc mutants with tailored selectivity for FcγRs, FcRn, C1q, and other Fc-binding ligands.

### Directed evolution identifies glycosylated Fc variants with markedly improved hFcγRIIIa selectivity

**Fig. 2:**
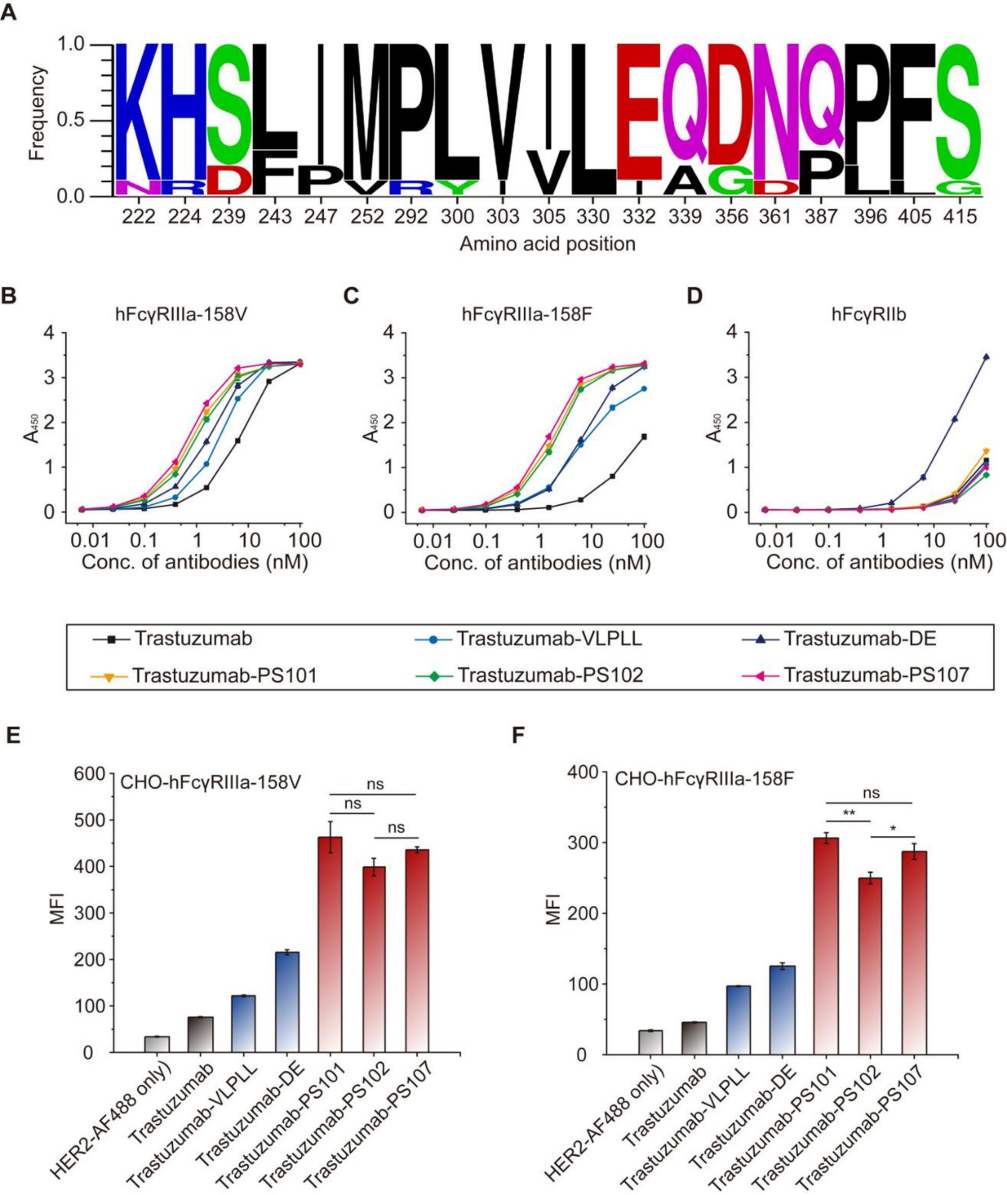
Identification of Fc variants with enhanced FcγRIIIa/FcγRIIb binding selectivity. (**A**) Sequence logo plot of enriched Fc variants isolated by flow cytometric sorting. (**B–D**) ELISA-based binding analyses of Fc variants to hFcγRIIIa-158V (**B**), hFcγRIIIa-158F (**C**), and hFcγRIIb (**D**). (**E**,** F**) Bar graphs showing the median fluorescence intensity (MFI) of trastuzumab-Fc variants binding to CHO cell displaying hFcγRIIIa-158V (**E**) and CHO-hFcγRIIIa-158F (**F**). Error bars represent the standard deviation (SD) from three independent replicates. Statistical significance was determined using a two-tailed unpaired student’s t-test. ns, not significant (*p* > 0.05); *p ≤ 0.05; **p ≤ 0.01

To identify Fc variants exhibiting highly selective binding to the activating receptor hFcγRIIIa over the inhibitory hFcγRIIb, we employed our glycosylation-competent CHO surface display system to screen a combinatorial Fc variant library. The CHO library cells were incubated with 5 nM tetrameric hFcγRIIIa–streptavidin–AF647, 20 nM non-fluorescent tetrameric hFcγRIIb–streptavidin, and 1,000-fold diluted Protein A–FITC, followed by fluorescence-activated cell sorting (FACS) of the top ~ 2% AF647⁺/FITC⁺ population exhibiting preferential hFcγRIIIa binding and minimal hFcγRIIb recognition (Supplementary Fig. 3A). From these enriched cells, ten distinct Fc clones were recovered via PCR from integrated genomic DNA. Sequence analysis revealed convergent mutations at multiple amino acid residues, including several sites previously reported to influence FcγR binding. Among these, high-frequency substitutions were identified at F243L, P247I, R292P, Y300L, V305I, A330L, I332E, A339Q, and P387Q, underscoring these positions as critical hot spots for hFcγRIIIa selectivity engineering (Fig. 2A and Supplementary Fig. 3B).

Each variant was introduced into the trastuzumab framework for validation. ELISA-based assays showed that three variants, namely PS101, PS102, and PS107, conferred markedly improved binding to both hFcγRIIIa allotypes (158V and 158F), while maintaining minimal binding to hFcγRIIb (Fig. 2B–D). Notably, trastuzumab-PS variants exhibited superior hFcγRIIIa/hFcγRIIb selectivity relative to benchmark Fc designs VLPLL (MacroGenics) and DE (Xencor). Flow cytometric analysis using CHO cells expressing hFcγRIIIa-158V or -158F further confirmed enhanced functional engagement of PS variants with the activating hFcγRs, with significantly higher signal intensities than trastuzumab-VLPLL and trastuzumab-DE (Fig. 2E and F). BLI revealed that PS101 and PS107 achieved 262-fold (K_D_ = 1.07 nM vs. 280 nM) and 497-fold (K_D_ = 1.80 nM vs. 895 nM) affinity improvements for hFcγRIIIa-158V and − 158F, respectively, compared to wild-type trastuzumab (Table 1 and Supplementary Fig. 4). In contrast, binding to hFcγRIIb was reduced by up to 4.2-fold, substantially expanding the activating-to-inhibitory (A/I) selectivity window. The PS107 variant achieved A/I selectivity ratios of 1,103 (158V) and 2,096 (158F), surpassing wild-type (1.0), DE (2.1 and 2.3), and VLPLL (19.4 and 33.6).

**Table 1:** Measurement of equilibrium binding constants

	hFcγRIIIa-158V				hFcγRIIIa-158F				hFcγRIIb				A/I ratio	
	K_D_ (nM)	k_on_ (10^4^/Ms)	k_off_ (10^− 3^/s)	Fold	K_D_ (nM)	k_on_ (10^4^/Ms)	k_off_ (10^− 3^/s)	Fold	K_D_ (nM)	k_on_ (10^4^/Ms)	k_off_ (10^− 2^/s)	Fold	158V	158F
Trastuzumab	280 ± 10.0	Steady-state		1.00	895 ± 205	Steady-state		1.00	1020 ± 80.00	Steady-state		1.00	1.0	1.0
Trastuzumab-VLPLL	38.2 ± 4.72	2.82 ± 0.045	108 ± 11.6	7.33	70.6 ± 1.65	2.55 ± 0.028	180 ± 6.20	12.7	2700 ± 300.0	Steady-state		0.38	19.4	33.6
Trastuzumab-DE	2.04 ± 0.44	3.68 ± 0.11	7.51 ± 0.38	137	6.00 ± 0.32	3.23 ± 0.12	19.4 ± 0.31	149	15.92 ± 0.400	6.93 ± 0.019	110 ± 3.15	64.1	2.1	2.3
Trastuzumab-PS101	1.07 ± 0.12	3.07 ± 0.063	3.26 ± 0.30	262	3.05 ± 0.18	2.89 ± 0.028	8.79 ± 0.59	293	2300 ± 100.0	Steady-state		0.44	590.1	661.7
Trastuzumab-PS102	1.18 ± 0.16	3.31 ± 0.14	3.90 ± 0.35	237	2.15 ± 0.059	3.63 ± 0.042	7.79 ± 0.12	416	4300 ± 700.0	Steady-state		0.24	1000.3	1754.9
Trastuzumab-PS107	1.07 ± 0.0065	3.38 ± 0.17	3.62 ± 0.20	262	1.80 ± 0.17	2.92 ± 0.020	5.25 ± 0.54	497	4300 ± 200.0	Steady-state		0.24	1103.2	2096.1

To determine whether PS variants preserved binding to other immunologically relevant receptors. ELISA confirmed comparable binding to hFcγRI, hFcγRIIa-131H, hFcγRIIa-131R, and hFcRn (both pH 6.0 and pH 7.4) relative to wild-type Fc (Supplementary Fig. 5). Given overlapping Fc contact regions among hFcγRI, hFcγRIIa, and hFcγRIIb, these results indicate that PS variants retain receptor compatibility while selectively enhancing hFcγRIIIa affinity. These features position PS101, PS102, and PS107 as promising Fc scaffolds for next-generation therapeutic antibodies with optimized effector function profiles.

### PS variants outperform clinically validated Fc designs in enhancing trastuzumab effector functions across HER2-positive tumors

Prior to assessing effector function, we first confirmed that Fc engineering did not compromise antigen binding. Flow cytometry analysis using SK-BR-3 cells revealed that all trastuzumab variants incorporating VLPLL, DE, PS101, PS102, and PS107 maintained HER2 binding comparable to wild-type trastuzumab, confirming preservation of antigen recognition despite Fc modifications (Supplementary Fig. 6).

**Fig. 3:**
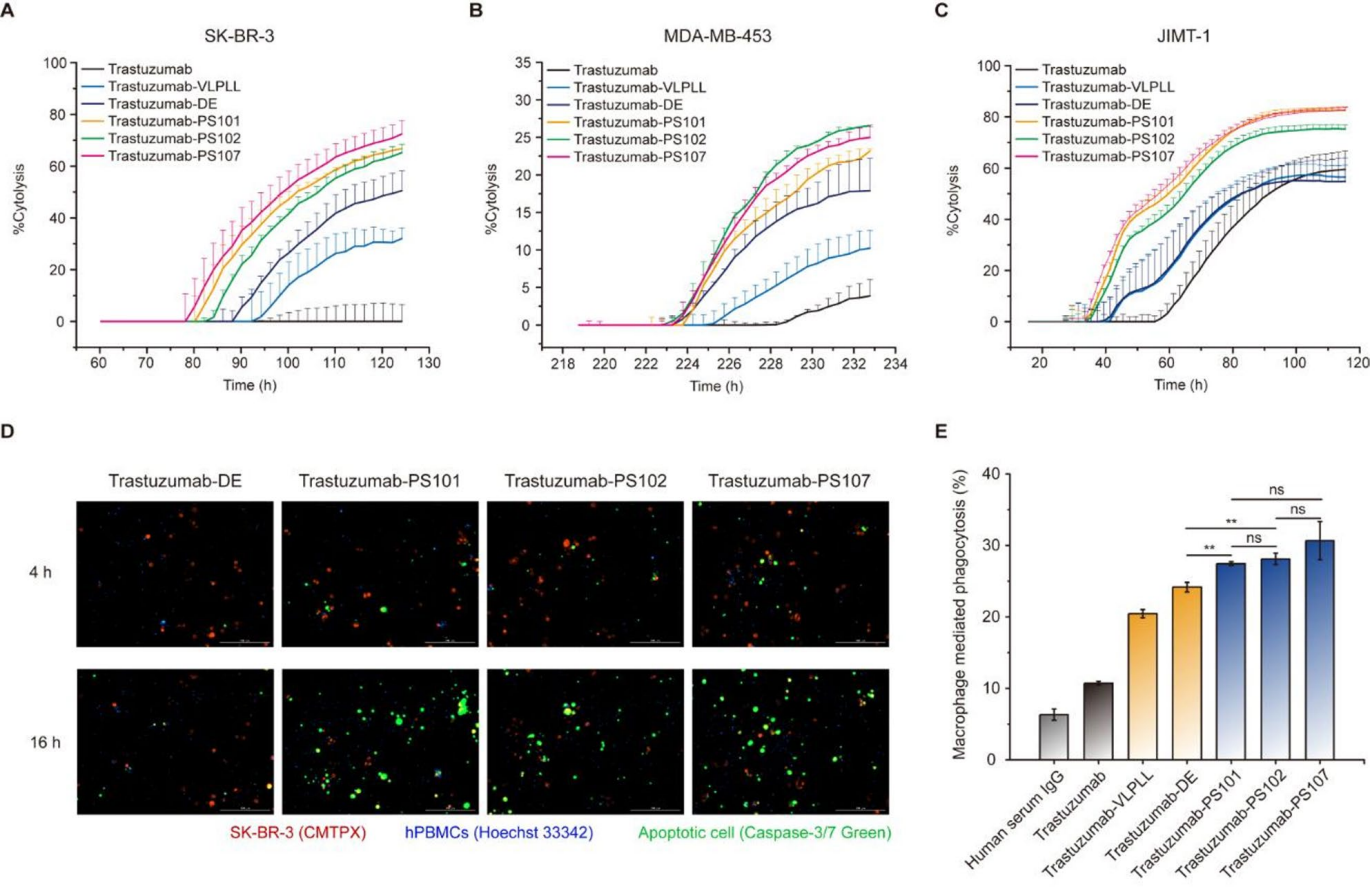
In vitro functional evaluation of trastuzumab-Fc variants. **(A–C)** Real-time cytotoxicity (%) of trastuzumab and trastuzumab-Fc variants (trastuzumab-VLPLL, -DE, -PS101, -PS102, and -PS107) assessed using PBMCs and human breast cancer cell line with high (SK-BR-3, 100 pM of antibodies, E:T = 5:1, *FCGR3A-158V/F* heterozygote, **A**), intermediate (MDA-MB-453, 20 pM of antibodies, E:T = 2:1, *FCGR3A-158V/F* heterozygote, **B**) or low (JIMT-1, 20 pM of antibodies, E:T = 10:1, *FCGR3A-158V/V* homozygote, **C**) HER2 expression. (**D**) Live-cell imaging analysis of ADCC activity induced by trastuzumab and Fc-engineered variants against SK-BR-3. SK-BR-3 cells were stained with CMTPX, PBMCs with Hoechst 33342, and apoptotic cells with Caspase-3/7 Green Detection Reagent. (**E**) Analysis of ADCP activity of trastuzumab and trastuzumab-Fc variants using monocyte-derived macrophage (*FCGR2A-131H/H* homozygote) and SK-BR-3 cells. Statistical significance was determined using a two-tailed unpaired student’s t-test. ns, not significant (*p* > 0.05); *p ≤ 0.05; **p ≤ 0.01

We next evaluated cell-based potency of each variant across multiple HER2-positive cancer cell lines with varying levels of HER2 surface expression (Supplementary Fig. 7). In SK-BR-3 cells, which express high HER2 levels, real-time cytotoxicity assays showed that trastuzumab-PS101, -PS102, and -PS107 induced significantly greater target cell lysis than trastuzumab counterparts bearing wild-type, VLPLL, or DE Fc variant (Fig. 3A and Supplementary Fig. 8A). Similarly, in MDA-MB-453 cells with intermediate HER2 expression, PS-containing antibodies also outperformed all controls (Fig. 3B and Supplementary Fig. 8B). In the trastuzumab-refractory JIMT-1 cell line, characterized by low HER2 expression, trastuzumab PS variant maintained potent cytotoxic activity, whereas wild-type and benchmark variants elicited minimal to modest responses (Fig. 3C and Supplementary Fig. 8C). Beyond enhancing potency, PS variants also accelerated cytotoxic kinetics. Impedance-based real-time cytotoxicity assays revealed earlier onset and higher endpoint lysis by trastuzumab PS variants compared to benchmark Fc-engineered formats (Fig. 3A-C). Live-cell imaging of PBMC co-cultures corroborated these findings, showing minimal differences at 4 h but markedly greater SK-BR-3 cell lysis by PS variants at 16 h (Fig. 3D), highlighting their capacity to intensify cytotoxic responses during prolonged effector engagement.

We further assessed antibody-dependent cellular phagocytosis (ADCP) using macrophages derived from human PBMCs. Flow cytometric quantification revealed that PS101, PS102, and PS107 conferred markedly enhanced macrophage-mediated phagocytosis of SK-BR-3 cells compared to wild-type, VLPLL, or DE (Fig. 3E). Since hFcγRIIa binding was preserved across all variants, the improved ADCP is attributable to enhanced hFcγRIIIa engagement—consistent with previous evidence linking this receptor to macrophage activation [29]. Together, these findings establish that PS variants significantly outperform clinically validated Fc designs in both ADCC and ADCP without compromising HER2 binding or hFcγRIIa compatibility. Their robust potency across HER2^+^ tumor models, including those resistant to trastuzumab, highlights their clinical promise as next-generation Fc-engineering modules.

### PS variants improve cytotoxic potency across diverse approved antibody scaffolds

**Fig. 4:**
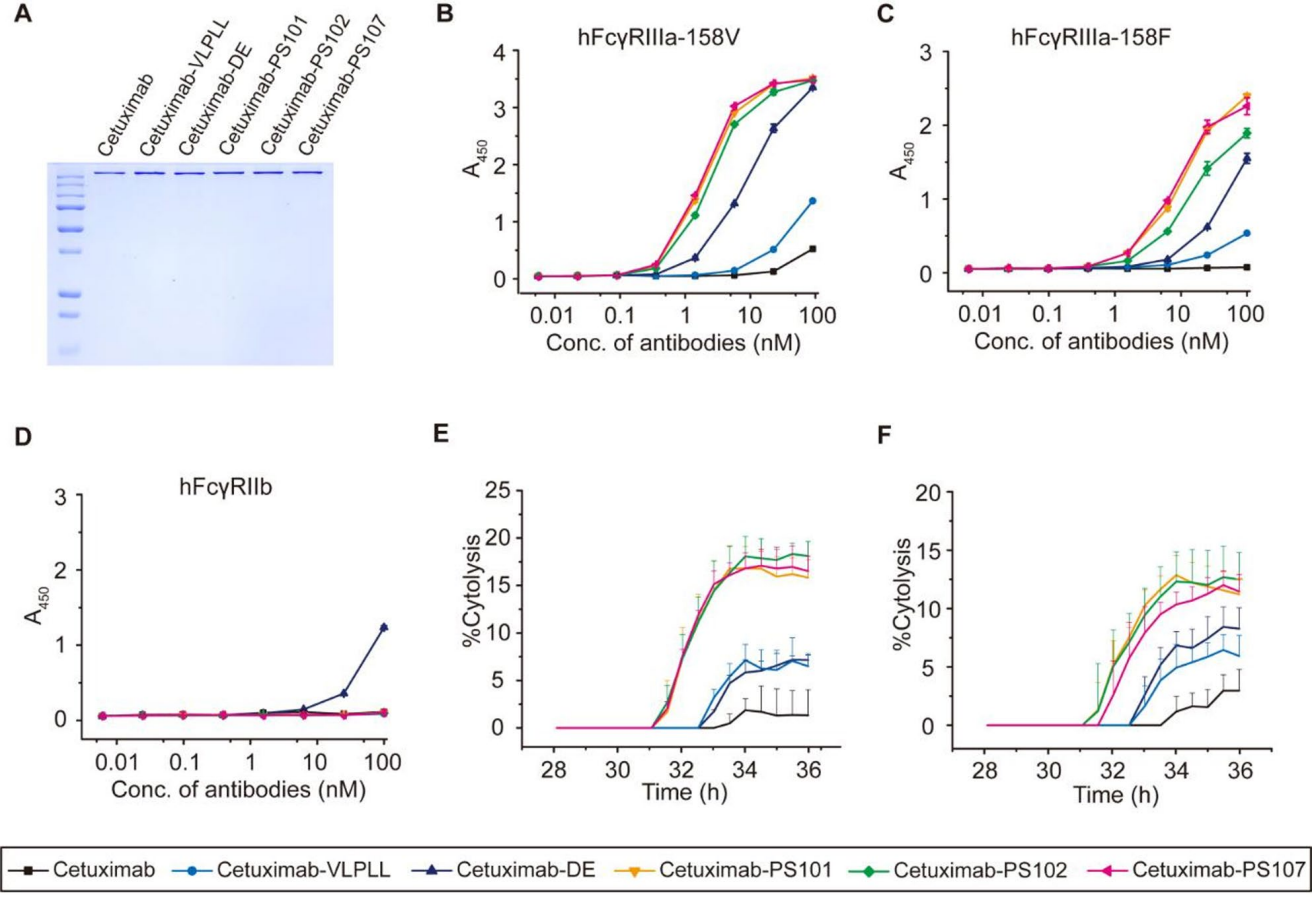
Applicability of PS Fc variants to cetuximab and enhancement of ADCC effector functions. (**A**) SDS-PAGE analysis of cetuximab and Fc-engineered variants (cetuximab-VLPLL, -DE, -PS101, -PS102, and -PS107) expressed in Expi293F cells and purified by Protein A affinity chromatography. (**B–D**) ELISA-based analysis of cetuximab and cetuximab-Fc variants. Binding to hFcγRIIIa-158V (**B**), hFcγRIIIa-158F (**C**), hFcγRIIb (**D**) was measured. (**E**,** F**) Real-time ADCC activity induced by cetuximab and cetuximab-Fc variants using freshly isolated human PBMCs as effector cells and EGFR-overexpressing A431 epidermoid carcinoma cells as targets. (**E**) E:T ratio of 5:1 with 20 pM antibody. (**F**) E:T ratio of 2:1 with 100 pM antibody

To rigorously evaluate the translational versatility of PS variants, we introduced PS101, PS102, and PS107 into two clinically approved antibody therapeutics: cetuximab (anti-EGFR), primarily employed as a first-line therapy for metastatic colorectal and head-and-neck squamous cell carcinomas, and rituximab (anti-CD20), widely utilized as a frontline treatment for non-Hodgkin’s B-cell lymphomas. ELISA-based hFcγR profiling of cetuximab-PS variants confirmed retained binding to hFcγRI, hFcγRIIa-131H, hFcγRIIa-131R, and hFcRn (pH 6.0), comparable to wild-type cetuximab, with markedly increased binding to hFcγRIIIa-158V and − 158F and complete loss of detectable interaction with hFcγRIIb (Supplementary Fig. 9 and Fig. 4A–D). Real-time cytotoxicity assays using EGFR-overexpressing A431 carcinoma cells and PBMC effectors revealed that cetuximab-PS101, -PS102, and -PS107 conferred significantly enhanced lysis of target cells relative to wild-type, VLPLL- or DE-engineered cetuximab at both 5:1 and 2:1 effector-to-target ratios (Fig. 4E–F and Supplementary Fig. 10). These results demonstrate the compatibility of PS variants with the cetuximab scaffold and their capacity to boost hFcγRIIIa-mediated cytotoxic function while preserving interactions with hFcγRIIa or hFcRn.

**Fig. 5:**
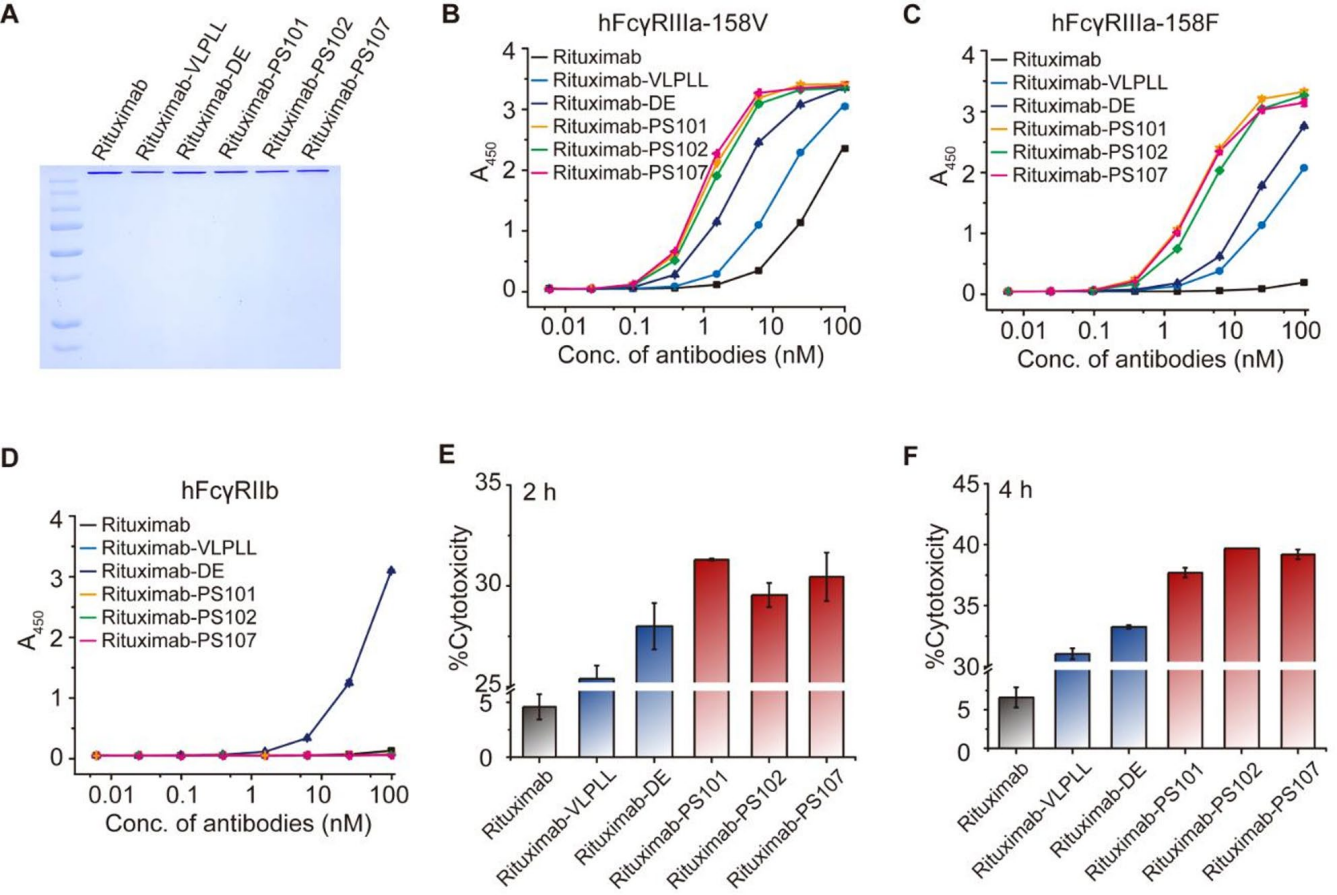
Extension of PS Fc variants to rituximab and enhancement of ADCC activity. (**A**) SDS-PAGE showing purified rituximab and Fc-engineered variants (rituximab-VLPLL, -DE, -PS101, -PS102, and -PS107) expressed in Expi293F cells and purified by Protein A affinity chromatography. (**B–D**) Receptor binding profiles of rituximab and its Fc-engineered counterparts determined by ELISA. Interactions with hFcγRIIIa-158V (**B**), hFcγRIIIa-158F (**C**), and hFcγRIIb (**D**) were analyzed across a range of antibody concentrations. (**E**,** F**) ADCC activity mediated by rituximab and Fc-engineered rituximab variants (rituximab-VLPLL, -DE, -PS101, -PS102, and -PS107) against CD20-expressing Ramos B lymphoma cells using GFP-CD16-V/V-NK92 cells as effector cells. Cytotoxic responses were measured at an antibody concentration of 50 pM after 2 h (**E**) or 4 h (**F**) of co-incubation

We next extended this approach to rituximab. hFcγR profiling of rituximab-PS variants similarly showed preserved binding to hFcγRI, hFcγRIIa-131H, hFcγRIIa-131R, and hFcRn, along with significantly elevated binding to hFcγRIIIa-158V and − 158F and undetectable binding to hFcγRIIb (Supplementary Fig. 11 and Fig. 5A–D). In GFP-CD16-V/V-NK92-mediated ADCC assays using CD20⁺ B-cell lymphoma targets, rituximab-PS101, -PS102, and -PS107 elicited markedly greater cytolysis (31.3%, 29.6%, and 30.5%, respectively) than wild-type rituximab (4.6%), and outperformed rituximab-VLPLL (25.4%) and rituximab-DE (28.0%). By the final time point, cytolysis further increased to 37.7%, 39.7%, and 39.2%, respectively, whereas rituximab-VLPLL and rituximab-DE reached 31.1% and 33.3% (Fig. 5E–F). These data underscore the robust enhancement conferred by PS variant integration. Collectively, these findings establish PS101, PS102, and PS107 as potent and versatile Fc modules that reliably augment hFcγRIIIa-driven effector function across distinct antibody scaffolds. Their consistent superiority over benchmark-engineered Fc formats affirms their value as a broadly applicable strategy for designing next-generation antibody therapeutics.

### PS variants demonstrate cross-species FcγRIII engagement, supporting preclinical translation

**Fig. 6:**
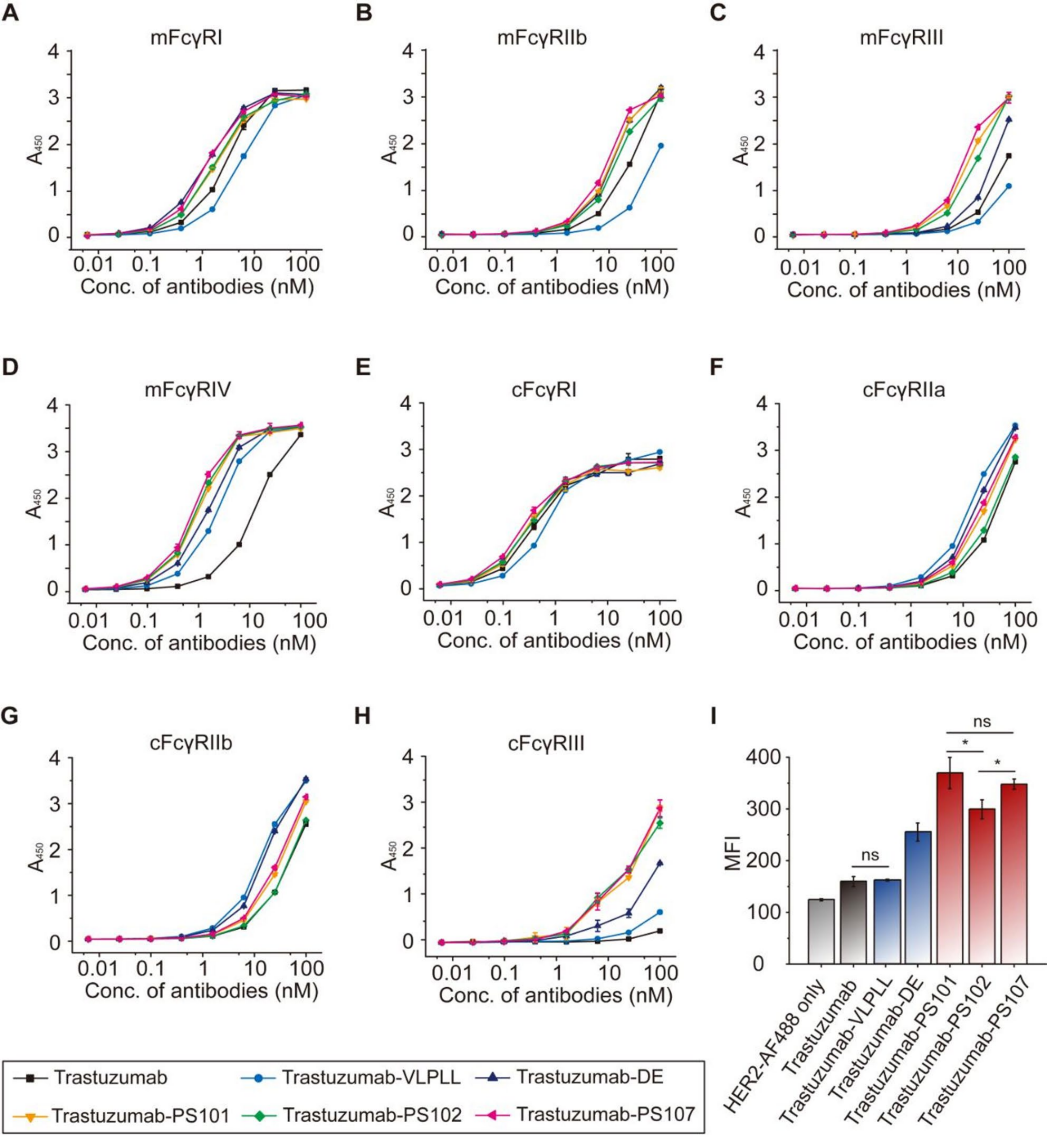
Analysis of cross-species FcγRs binding. (**A–D**) ELISA-based binding assays of trastuzumab and its Fc variants (trastuzumab-VLPLL, -DE, -PS101, -PS102, and -PS107) to mouse FcγRs: mFcγRI (**A**), mFcγRIIb (**B**), mFcγRIII (**C**), and mFcγRIV (**D**). (**E–H**) Binding activities of trastuzumab and its Fc variants to cFcγR: cFcγRI (**E**), cFcγRIIa (**F**), cFcγRIIb (**G**), and cFcγRIII (**H**). Error bars indicate values from duplicate experiments. (**I**) Bar graph showing binding of trastuzumab and its Fc variants to CHO cells expressing cFcγRIII. Error bars represent SD from three independent replicates. Statistical significance was determined using a two-tailed unpaired student’s t-test. ns, not significant (*p* > 0.05); *p ≤ 0.05

To facilitate the translational development of antibodies incorporating PS variants, we comprehensively evaluated their cross-species Fc receptor binding using mouse and cynomolgus monkey, two species widely employed for modeling antibody efficacy and pharmacokinetics in preclinical settings. Recombinant dimeric Fc receptors from mouse (mFcγRI, mFcγRIIb, mFcγRIII, mFcγRIV, and mFcRn) and cynomolgus monkey (cFcγRI, cFcγRIIa, cFcγRIIb, cFcγRIII, and cFcRn) were produced as GST-tagged fusion proteins in Expi293F cells and purified for ELISA-based binding assays (Supplementary Fig. 12). Among the murine orthologs, mFcγRIV and mFcγRIII share approximately 67% and 48% sequence identity with hFcγRIIIa, respectively [30]. mFcγRIV is predominantly expressed on monocytes and macrophages, whereas mFcγRIII is the only FcγR expressed on murine NK cells [31]. These orthologs serve as key surrogates for assessing Fc-dependent immune function in murine systems. Trastuzumab variants incorporating PS101, PS102, or PS107 consistently demonstrated increased binding to both mFcγRIII and mFcγRIV compared to wild-type, VLPLL, and DE variants, recapitulating their enhanced hFcγRIIIa engagement (Fig. 6A–D). Binding to mFcRn at acidic pH (6.0) was maintained at levels comparable to wild-type, whereas DE exhibited a modest reduction (Supplementary Fig. 13).

In the cynomolgus monkey FcγR panel, trastuzumab variants containing PS101, PS102, or PS107 also showed markedly enhanced binding to cFcγRIII, which shares more than 90% sequence identity with hFcγRIIIa [30]. In contrast, all trastuzumab variants—including wild-type, VLPLL, DE, and PS-containing constructs—demonstrated similar binding to cFcγRI, cFcγRIIa, and cFcγRIIb (Fig. 6E–H). Flow cytometric analysis using CHO cells stably expressing cFcγRIII further confirmed that PS variants enable greater recognition of FcγRIII orthologs across species, including mFcγRIII, mFcγRIV, and cFcγRIII (Fig. 6I). Furthermore, PS variants retained pH-dependent binding to cFcRn comparable to wild-type trastuzumab, while DE showed a slight decrease (Supplementary Fig. 14).

We next examined cetuximab-based constructs. PS101, PS102, and PS107 exhibited significantly enhanced binding to mFcγRIV compared to wild-type cetuximab, along with improved binding to mFcγRI (Supplementary Fig. 15A–D). mFcRn binding was maintained in all PS variants and indistinguishable from wild-type levels, whereas DE again exhibited reduced interaction (Supplementary Fig. 15E–F). In cynomolgus monkey assays, PS variants conferred higher binding to cFcγRIII relative to wild-type, VLPLL, and DE. Notably, cFcγRI binding was reduced in the VLPLL variant but remained similar to wild-type in the PS and DE constructs (Supplementary Fig. 16). Consistent patterns emerged in rituximab-based formats, where PS101, PS102, and PS107 enhanced binding to both mFcγRIV and mFcγRI relative to wild-type rituximab, with binding levels comparable to DE (Supplementary Fig. 17A–D). All three PS variants also demonstrated substantially increased binding to cFcγRIII, while VLPLL exhibited reduced binding to cFcγRI. PS and DE variants retained binding affinity to cFcγRI comparable to wild-type levels (Supplementary Fig. 18A–D). pH-dependent binding to cFcRn was preserved across all rituximab variants (Supplementary Fig. 18E–F). Together, these results establish that PS101, PS102, and PS107 robustly enhance FcγRIII binding across murine and primate orthologs while retaining favorable compatibility with FcγRIIa and FcRn. This cross-species reactivity highlights the versatility of PS variants as a generalizable platform for Fc engineering, supporting both preclinical efficacy modeling and clinical translational effector function-dependent antibody therapeutics.

### PS variants confer potent effector-driven tumor suppression to trastuzumab in an in vivo trastuzumab-resistant xenograft model

To investigate the therapeutic potential of PS variants in trastuzumab-refractory settings, we first analyzed the pharmacodynamic profiles of trastuzumab and its Fc-engineered derivatives in vivo. Serum half-life measurements were conducted in hFcRn transgenic (Tg) mice following intravenous injection. All trastuzumab-Fc variants incorporating FcγRIIIa-enhancing mutations, including DE, PS101, and PS107, exhibited shortened circulating half-lives relative to wild-type trastuzumab, despite maintaining pH-dependent binding to hFcRn at pH 6.0. Although DE showed a slight reduction in hFcRn binding, PS101 and PS107 retained hFcRn affinity comparable to wild-type. These results indicate that the abbreviated half-lives are not due to impaired hFcRn engagement but rather reflect enhanced FcγR-mediated clearance, particularly through mFcγRIV (Supplementary Tables 3 and Supplementary Fig. 19). To dissect the role of FcγR-mediated clearance, we employed hFcRn Tg mice on an NSG background lacking T, B, and NK cells (NSG FcRn^−/−^ hFcRn Tg mice). To approximate the physiological environment of competitive hFcRn occupancy, mice were preloaded with 500 mg/kg of pooled human IgG three days prior to antibody administration. In this immune-deficient context, PS101, PS107, and DE exhibited comparable half-lives (Supplementary Tables 4 and Supplementary Fig. 20), confirming that FcγR-bearing effector cells primarily mediate the enhanced clearance observed in immune-competent animals. Since innate immune cells such as monocytes and macrophages persist in this model, residual differences in half-life likely reflect clearance via mFcγRIII and mFcγRIV. Additionally, competition from excess human IgG reduced recycling efficiency, contributing to shorter half-lives relative to those observed in settings without IgG preloading.

**Fig. 7:**
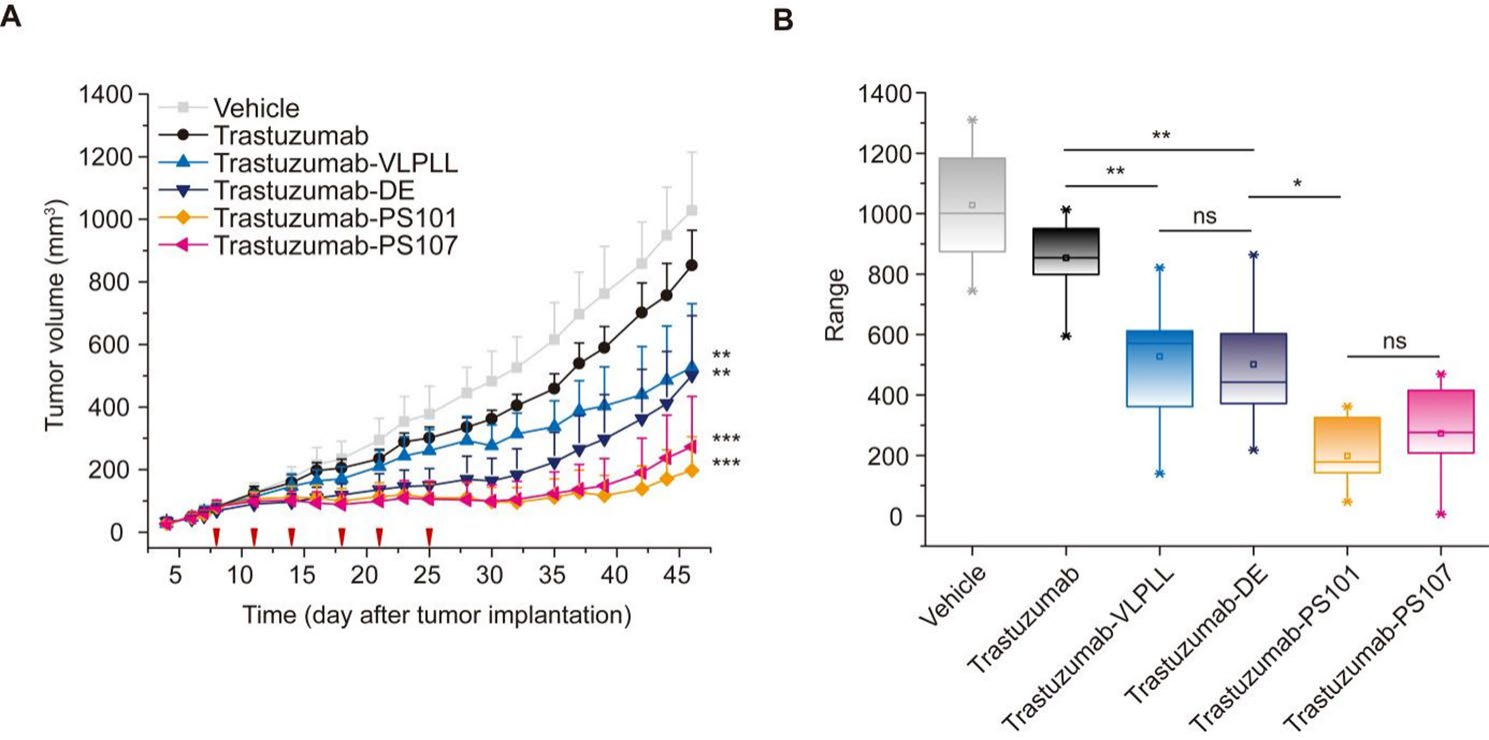
Evaluation of tumor suppression effects of Fc variants in a nude mouse xenograft model. (**A**) Anti-tumor efficacy of trastuzumab and Fc-engineered trastuzumab variants in JIMT-1 xenograft-bearing BALB/c nude mice (*n* = 10 per group). Red arrows indicate antibody administration time points. Statistical significance:, ** (*p* ≤ 0.01), *** (*p* ≤ 0.001) versus trastuzumab group. (**B**) Tumor volume range on day 46 post-implantation. Tumor volume was calculated using the formula (length × width²)/2, where L is tumor length and W is tumor width. Statistical significance was determined using a one-way ANOVA. ns, not significant (*p* > 0.05); *p ≤ 0.05; **p ≤ 0.01

We next evaluated therapeutic efficacy in a trastuzumab-resistant xenograft model. BALB/c nude mice were implanted subcutaneously with JIMT-1, a HER2-positive breast carcinoma cell line known to exhibit reduced responsiveness to trastuzumab. Once tumors reached a volume of approximately 80 mm³, mice (*n* *= 10* per group) received intraperitoneal injections of vehicle, trastuzumab, or trastuzumab-Fc variants every four days for a total of six doses, with tumor growth monitored over 46 days (Fig. 7). Trastuzumab-PS101 and -PS107, which exhibited the highest binding to both mFcγRIII and mFcγRIV, elicited the most pronounced tumor suppression. Remarkably, two mice in the PS107-treatment group experienced near-complete tumor regression, suggesting sustained and potent anti-tumor responses (Supplementary Fig. 21). These data highlight that enhanced effector engagement can overcome resistance and confer robust therapeutic benefit, even when associated with moderately reduced systemic half-life. Trastuzumab-DE, with intermediate affinity for mFcγRIII and mFcγRIV, produced moderate tumor inhibition but was associated with rapid tumor regrowth in the latter phase of the study, indicative of limited immune activation and insufficient durability. VLPLL, which increased mFcγRIV but not mFcγRIII binding, initially performed similarly to wild-type trastuzumab but showed progressive tumor suppression during later phases, eventually surpassing trastuzumab efficacy. These findings underscore the critical importance of co-engaging both mFcγRIII and mFcγRIV, which share overlapping effector expression and functional roles with human FcγRIIIa, to achieve durable in vivo therapeutic responses.

Taken together, our results demonstrate that glycosylation-competent mammalian surface display enables the discovery of Fc variants capable of converting suboptimal antibodies into highly effective therapeutics by enhancing effector cell engagement. The absence of significant body weight loss across all treatment groups (Supplementary Fig. 21H) further underscores the safety and translational potential of PS variants as next-generation Fc engineering modules for antibody therapeutics.

## Discussion

We established a glycosylation-competent CHO surface display platform that integrates mammalian post-translational fidelity with high-throughput screening capabilities, enabling precise engineering of Fc variants with tailored receptor engagement profiles. Fc–FcγRIIIa interactions are highly sensitive to N297 glycan composition; previous studies have demonstrated that the binding affinity can vary up to two orders of magnitude solely as a function of glycan structure, even when the polypeptide backbone is identical [32]. Moreover, high-mannose glycoforms derived from yeast have been reported to exhibit altered interactions with all human FcγRs, leading to changes in antibody effector function. These findings highlight the importance of screening Fc variants under physiologically relevant glycosylation conditions. Leveraging the human-like glycosylation capacity and industrial relevance of CHO cells, this platform enables direct selection of FcγR-binding variants within a native-like glycan environment. Through combinatorial mutagenesis integrated with glycan-competent display, we identified Fc domains exhibiting markedly enhanced receptor affinity and selectivity, culminating in potent NK cell-mediated ADCC.

Incorporation of PS variants into therapeutic antibodies including trastuzumab, rituximab, and cetuximab consistently improved cytotoxic function, as confirmed by real-time effector assays. In murine xenografts, trastuzumab-PS variants with enhanced Fc-mFcγR interactions significantly improved tumor control, outperforming benchmark Fc-engineered formats. Importantly, these enhancements occurred without concomitant increase in FcγRIIb binding, indicating a receptor-selective rather than promiscuous affinity profile. Whereas DE and VLPLL originated from structure-based in silico screening and yeast display in non-native glycosylation contexts, respectively, our system directly selects Fc variants in native-like glycosylation environments, ensuring physicochemical and functional compatibility. In addition to improved tumor control, PS variants exhibited reduced systemic persistence in hFcRn Tg mice, consistent with enhanced FcγR–mediated clearance. In certain therapeutic contexts, shorter systemic exposure may help limit prolonged immune activation and associated off-target toxicities, as sustained immune stimulation has been linked to immune-related adverse events in immunotherapy settings such as immune checkpoint blockade [33]. Pharmacokinetic properties are therefore context-dependent and may be tuned according to therapeutic needs. Future studies may explore combining PS substitutions with FcRn–binding-enhancing mutations, such as YTE [34], LS [35], PFc29 [36], or YML [37], to extend serum persistence while preserving receptor selectivity.

PS variants represent the most potent hFcγRIIIa binders reported to date among Fc-engineered formats for ADCC. Their robust engagement of both hFcγRIIIa allotypes (158V and 158F) ensures genotype-independent efficacy across patient populations. This property is particularly relevant because multiple clinical studies with therapeutic IgG1 antibodies–including trastuzumab [13], cetuximab [15], and rituximab [14]–have demonstrated that patients carrying the low-affinity F/F genotype often exhibit reduced clinical responses compared with those harboring the high-affinity V/V genotype. Simultaneously, minimal engagement of hFcγRIIb shifts the activating-to-inhibitory FcγR balance toward productive effector signaling. FcγRIIb is frequently upregulated in the tumor microenvironment, particularly on tumor-associated macrophages, leading to a reduced A/I ratio that constrains antibody-mediated effector function [38–40]. In humanized FcγR models using anti-CTLA-4 antibodies, reducing FcγRIIb engagement restored intratumoral Treg depletion and enhanced anti-tumor efficacy. Accordingly, the diminished FcγRIIb binding of the PS variants may help preserve effective effector activity in inhibitory FcγR-rich environments. This receptor-selective profile distinguishes PS variants from glycoengineering approaches, such as afucosylation, which primarily enhance the affinity of FcγRIIIa without significantly reducing the engagement of FcγRIIb. Through Fc engineering, PS variants enable the concurrent enhancement of binding to activating receptors and minimization of inhibitory signaling, providing more precise control of the balance of effectors. Beyond selective FcγRIIIa binding, PS variants enhanced both NK cell-mediated ADCC and macrophage-driven ADCP. Given the substantial heterogeneity of immune infiltration in solid tumors, where lymphoid and myeloid populations can vary across tumor types and even within different tumor regions [41, 42], this dual activation could be particularly advantageous. By engaging both cytotoxic and phagocytic pathways, PS variants may broaden antitumor efficacy in complex microenvironments.

Our strategy also offers distinct advantages over alternative NK cell engaging approaches. Several bispecific killer engagers (BiKEs) and trispecific killer engagers (TriKEs), including IL-15–armed constructs, have been developed to co-engage hFcγRIIIa and tumor antigens via non-natural antibody architectures [43–46]. Additionally, Affimed’s Redirected Optimized Cell Killing (ROCK) platform exemplifies non-canonical NK cell engagers that mediate cytotoxicity using bispecific scaffolds [47, 48]. These synthetic platforms often require extensive optimization to achieve clinical-grade properties, particularly regarding physicochemical stability and pharmacokinetics. In contrast, PS variants preserve the natural IgG1 format while achieving selective, high-affinity hFcγRIIIa engagement—a design advantage that circumvents the structural and pharmacologic complexities faced by non-natural NK cell engagers. This native-format compatibility facilitates manufacturing, streamlines regulatory pathways, and allows seamless integration into diverse antibody modalities including isotype switching, bispecifics, Fc-fusions, and immune-modulating conjugates.

A recent phase 1 study demonstrated that precomplexing allogeneic NK cells with AFM13, a bispecific CD30 × CD16A innate engager, achieved a 92.9% overall response rate in patients with CD30⁺ lymphoma refractory to brentuximab vedotin and PD-1 blockade [49]. These results validate the clinical potential of NK cell–antibody complexes. Building on this precedent, PS variant–bearing IgG1s could similarly be preloaded onto adoptively transferred NK cells to generate targeted, modular cytotoxic cell therapies. Their minimized hFcγRIIb binding, strong engagement of both hFcγRIIIa allotypes, and structurally native IgG1 backbone further support their safety, functionality, and translational applicability.

Building on their receptor-level precision and NK cell–engaging potential, PS variants also provide a rational foundation for combination immunotherapy. Co-targeting inhibitory receptors such as NKG2A [50, 51], TIGIT [52, 53], or PD-1 [54, 55] alongside hFcγRIIIa engagement may amplify NK cell responses beyond monotherapy effects. Similarly, co-engagement of activating NK receptors like NKp30, NKp44, or NKp46 [56, 57] may drive convergent signaling cascades that potentiate degranulation, cytokine production, and tumor cell killing. Importantly, these synergistic strategies are fully compatible with the canonical IgG1 backbone, preserving developability and translational integration.

A particularly promising future direction lies in combining sequence-based engineering with glycoengineering strategies such as defucosylation. Since afucosylated Fc glycans increase hFcγRIIIa affinity and enhance ADCC, producing PS variants in FUT8-knockout CHO cell lines [58] would allow direct comparison of glycan- and sequence-mediated effects. Such studies could determine whether these mechanisms are additive or synergistic in promoting NK cell activation. To elucidate the structural underpinnings of enhanced receptor engagement conferred by PS107, we employed AlphaFold3 to model ternary complexes of wild-type Fc and PS107 with the two major hFcγRIIIa allotypes (158V and 158F; Supplementary Fig. 22). Consistent with the inherently asymmetric nature of Fc–FcγR interactions, the Fc B chain of Fc contributed disproportionately to receptor engagement. In PS107, the I332E substitution introduced novel hydrogen bonds in the 158V complex, whereas engagement with the 158F allotype shifted contact residues from G237 and D265 (wild-type) to G236 and E332, positions closer to the receptor interface, likely enhancing spatial complementarity. In addition, the A330L mutation created a distinct hydrophobic interaction with G89, a residue conserved in both allotypes. Taken together, these substitutions may act synergistically by combining improved polar contacts (I332E) with enhanced hydrophobic packing (A330L), thereby stabilizing both allotypes of FcγRIIIa engagement. This cooperative reshaping of the interface provides a mechanistic rationale for the enhanced binding affinity of PS107 across both allotypes. Nonetheless, experimental validation via X-ray crystallography or cryo-EM, complemented by dynamic conformational analyses such as HDX-MS or NMR, will be necessary to deconvolute the precise energetic contributions of each substitution.

From an immunological perspective, comprehensive transcriptomic and functional analyses of NK cell and myeloid cell populations exposed to Fc-engineered antibodies will be critical to delineate how tailored Fc–FcγR interactions reprogram effector cell states and modulate the tumor microenvironment. To evaluate potential immunogenicity risks, we conducted an in silico MHC class II binding analysis using the IEDB platform. Predicted binding affinities were assessed across 27 prevalent HLA alleles for wild-type Fc and its engineered counterparts (VLPLL, DE, PS101, PS102, and PS107). None of the engineered variants exhibited a significant elevation in predicted peptide-MHC class II binding compared to wild-type, suggesting that the introduced mutations are unlikely to generate neoepitopes recognized by CD4^+^ T cells (Supplementary Fig. 23). While experimental confirmation using primary human antigen-presenting cells remains necessary, these findings support a low immunogenicity risk profile and further reinforce the translational potential of PS variants.

## Conclusion

In conclusion, we present a glycosylation-competent mammalian display platform that enables precise selection of receptor-specific, functionally enhanced Fc variants within the native IgG1 context. By preserving glycan fidelity during selection and systematically optimizing sequence composition, this strategy overcomes key limitations in Fc engineering—namely, the challenge of enhancing effector potency while maintaining receptor selectivity and developability. The resulting variants demonstrate strong NK cell activation, robust tumor cytotoxicity, and broad adaptability to existing and emerging immunotherapeutic modalities. Collectively, our findings establish this platform as a structurally coherent and translationally robust strategy for advancing Fc-driven precision immunotherapy.

## Supplementary Information

Below is the link to the electronic supplementary material.

Supplementary Material 1

## Data Availability

The datasets generated during and/or analyzed during the current study are available from the corresponding author on reasonable request.
