# Supplementary material for: Precision Fc remodeling via glycosylation-competent CHO display enables ultra-selective FcγRIIIa targeting and enhanced antitumor activity
Source: J Biol Eng. 2026 Apr 1;20:89. doi: 10.1186/s13036-026-00671-8 (PMC13170290; doi:10.1186/s13036-026-00671-8)
Supplement: Supplementary file 1 — Supplementary Material 1 [file 13036_2026_671_MOESM1_ESM.pdf]

SUPPLEMENTARY INFORMATION

Precision Fc Remodeling via Glycosylation-Competent CHO Display Enables Ultra-Selective FcγRIIIa Targeting and Enhanced Antitumor Activity

Migyeong Jo[1,2,†], Suyeon Kim[2,3,†], Sanghwan Ko[3,††], Munsu Kyung[2,3], Seunghyeon Lee[1], Woo Hyung Ko[1], Wonju Lee[3,†††] and Sang Taek Jung[1,2,4,5,6,*]

[1]Department of Chemical and Biological Engineering, College of Engineering, Seoul National University, Seoul 08826, Republic of Korea

[2]Institute of Chemical Processes, Seoul National University, Seoul 08826, Republic of Korea

[3]Department of Biomedical Sciences, Graduate School, Korea University, Seoul 02841, Republic of Korea

[4]Interdisciplinary Program for Bioengineering, Seoul National University, Seoul 08826, Republic of Korea

[5]BioMAX, Seoul National University, Seoul 08826, Republic of Korea

[6]Seoul National University Medical Research Center (SNUMRC), Seoul 03080, Republic of Korea

†These authors contributed equally: Migyeong Jo and Suyeon Kim

††Current affiliation: Department of Chemical Engineering, University of Texas at Austin, Austin, TX 78712, USA

†††Current affiliation: Manufacturing Science & Technology Team, Manufacturing Science Group2, Samsung Bioepis, Incheon, Republic of Korea

*Correspondence to: Sang Taek Jung (stjung@snu.ac.kr)

Supplementary Materials and Methods

Reagents

Enzymes for molecular cloning, including Vent polymerase, restriction endonucleases, and T4 DNA ligase were obtained from New England Biolabs (Ipswich, MA, USA). Oligonucleotide primers were synthesized by Bionics (Seoul, Republic of Korea) and Cosmogenetech (Seoul, Republic of Korea). Affinity resins, including Ni-NTA agarose, Protein A agarose, and glutathione agarose 4B were sourced from Qiagen (Hilden, Germany), GenScript (Scotch Plains, NJ, USA), and Incospharm (Daejeon, Republic of Korea), respectively. For cell culture and transfection, Ham's F12 nutrient mix GlutaMAX, Opti-MEM reduced serum medium, Lipofectamine 2000, RPMI-1640 medium, GIBCO FreeStyle 293 expression medium were purchased from Thermo Fisher Scientific (Waltham, MA, USA). Fluorescent labeling kits (Alexa Fluor 647, FITC), an antibody (sheep anti-hC1q-polyclonal antibody-HRP conjugate), and key detection reagents (1-Step Ultra-TMB substrate solution, Hoechst 33342 trihydrochloride trihydrate, CellEvent™ caspase-3/7 detection reagents, and CellTracker™ red CMTPX dye) were also sourced from Thermo Fisher Scientific. Polyethyleneimine (PEI)-Max was sourced from Polysciences (Taipei, Taiwan). C1q protein and goat anti-GST-HRP conjugate were purchased from Quidel (San Diego, CA, USA) and Cytiva (Marlborough, MA, USA), respectively. The FAB2G biosensor for biolayer interferometry (BLI) analysis was obtained from Sartorius (Göttingen, Germany). APC-conjugated anti-human CD14 and anti-human CD11b antibodies were purchased from BioLegend (San Diego, CA, USA). Unless otherwise

specified, all other biochemical reagents were obtained from Sigma-Aldrich (St. Louis, MO, USA).

Construction of Plasmids

All plasmids and primers used in this study are summarized in table S1 and table S2. To generate CHO cell membrane-anchored Fc wild-type and Fc-T299L mutant, the corresponding genes (pcDNA5-Igκ-Fc-FLAG-PDGFR and pcDNA5-Igκ-Fc-TL-FLAG-PDGFR) were amplified using primers MJ#296/MJ#309 and template plasmids (pMAZ-IgH-GlycoT [1] and pMAZ-IgH rituximab-PFc29-TL [2]). The transmembrane domain of PDGFR was synthesized through primer assembly (MJ#256–259) and subseqeuntly amplified using MJ#310/MJ#272. These fragments were assembled using primers MJ#269/MJ#272, digested with *Nhe*I/*Hin*dIII endonucleases, and ligated into the pcDNA5-FRT vector.

For the construction of trastuzumab heavy chain variants, including VLPLL (L235V/F243L/R292P/Y300L/P396L), DE (S239D/I332E), PS101, PS102 and PS107, Fc genes for VLPLL and DE were synthesized by GenScript (Scotch Plains, NJ, USA), while Fc genes for PS101, PS102, and PS107 were obtained from recovered plasmids after sorting (pcDNA5-Igκ-Fc-PS101-FLAG-PDGFR, pcDNA5-Igκ-Fc-PS102-FLAG-PDGFR and pcDNA5-Igκ-Fc-PS107-FLAG-PDGFR, respectively). The Fc genes for VLPLL, DE, PS101 and PS107 were amplified using primers MJ#267/MJ#50, while the Fc gene for PS102 was amplified using primers MJ#616/MJ#50. The VH-CH1 region of the trastuzumab heavy chain was amplified using primers MJ#49/MJ#268 and the pMAZ-IgH-GlycoT template. The

amplified VH-CH1 and Fc variant gene fragments were then assembled using primers MJ#49/MJ#50, digested with *Bss*HII and *Xba*I, and ligated into the pMAZ-IgL [1] vector, generating pMAZ-IgH-trastuzumab-VLPLL, pMAZ-IgH-trastuzumab-DE, pMAZ-IgH-trastuzumab-PS101, pMAZ-IgH-trastuzumab-PS102, and pMAZ-IgH-trastuzumab-PS107. Similarly, heavy chain constructs for rituximab Fc variants (VLPLL, DE, PS101, PS102, and PS107) were generated by assembling the VH-CH1 fragment with the corresponding Fc fragment. The VH-CH1 gene of rituximab was PCR-amplified using primers MJ#545/MJ#546 with the pMAZ-IgH-rituximab [2] template. The Fc genes for VLPLL, DE, PS101, and PS107 were amplified using primers MJ#547/MJ#50, whereas the Fc gene for PS102 was amplified using primers MJ#548/MJ#50. The VH-CH1 and Fc fragments were assembled using primers MJ#545/MJ#50, digested with *Bss*HII and *Xba*I, and subsequently ligated into pMAZ-IgL [1] vector, generating the plasmids pMAZ-IgH-rituximab-VLPLL, pMAZ-IgH-rituximab-DE, pMAZ-IgH-rituximab-PS101, pMAZ-IgH-rituximab-PS102, and pMAZ-IgH-rituximab-PS107. The heavy chain and light chain of cetuximab (DrugBank #DB00002, pMAZ-IgH-cetuximab and pMAZ-IgL-cetuximab) were synthesized by GenScript (Scotch Plains, NJ, USA). Cetuximab heavy chain variants (VLPLL, DE, PS101, PS102, and PS107) were generated using assembly PCR following the same procedure. The VH-CH1 of cetuximab was amplified using the primers MJ#549/MJ#550 and the pMAZ-IgH-cetuximab template. The Fc genes for VLPLL, DE, PS101 and PS107 were amplified using the primers MJ#551/MJ#50, while the Fc gene for PS102 was amplified using primers MJ#552/MJ#50. Each prepared fragment was assembled using

primers MJ#549/MJ#50 and ligated into pMAZ-IgL [1] after *Bss*HII/*Xba*I digestion, yielding pMAZ-IgH-cetuximab-VLPLL, pMAZ-IgH-cetuximab-DE, pMAZ-IgH-cetuximab-PS101, pMAZ-IgH-cetuximab-PS102 and pMAZ-IgH-cetuximab-PS107.

For the construction of pMAZ-FcγRIIIa-158F-His and pMAZ-FcγRIIb-His, the FcγRIIIa-158F and FcγRIIb genes were PCR-amplified using pMAZ-FcγRIIIa-158F-GST [3] and pMAZ-FcγRIIb-GST [4] as templates, with primers MJ#573/MJ#574 and MJ#575/MJ#576, respectively. The PCR products were digested with *Bss*HII/*Xba*I restriction enzymes and subsequently ligated into the pMAZ-IgL [1] vector. Plasmids encoding the extracellular domains of mouse Fc receptors were synthesized by Twist Bioscience (South San Francisco, CA, USA) as follows: pMAZ-mFcγRI-GST (UniProt: P26151), pMAZ-mFcγRIIb-GST (UniProt: P08101), pMAZ-mFcγRIII-GST (UniProt: P08508), pMAZ-mFcγRIV-GST (UniProt: A0A0B4J1G0), pMAZ-mFcRn α chain-GST (UniProt: Q6PKB0), and pMAZ-mβ2m-GST (UniProt: P01887)). Similarly, plasmids encoding the extracellular domains of cynomolgus monkey Fc receptors were synthesized by Twist Bioscience as follows: pMAZ-cFcγRI-GST (UniProt: Q8SPW5), pMAZ-cFcγRIIa-GST (UniProt: Q8SPW4), pMAZ-cFcγRIIb-GST (UniProt: Q8SPW3), pMAZ-cFcγRIII-GST (UniProt: Q8SPW2), pMAZ-cFcRn α chain-GST (UniProt: Q8SPV9), and pMAZ-cβ2m-GST (UniProt: I7GKX8). Genes encoding CHO cell membrane-expressed hFcγRIIIa-158V, hFcγRIIIa-158F, and cFcγRIII were generated by PCR using primers MJ#565/MJ#566-567 for hFcγRIIIa-158V and hFcγRIIIa-158F, and primers MJ#602/MJ#603-604 for cFcγRIII. The template plasmids used for amplification were pMAZ-FcγRIIIa-158V-GST, pMAZ-FcγRIIIa-158F-GST,

and pMAZ-cFcγRIII-GST. The PCR-amplified products were digested with *Nhe*I/*Hin*dIII and ligated into the pcDNA5-FRT vector. All ligation products were subsequently transformed into *Escherichia coli* Jude1 [5] for plasmid propagation.

Expression and Purification of IgG Antibody-Fc Variants and Fc Receptors

For the expression of IgG antibodies-Fc variants, each IgG heavy chain plasmid (pMAZ-IgH-GlycoT, pMAZ-IgH-trastuzumab-VLPLL, pMAZ-IgH-trastuzumab-DE, pMAZ-IgH-trastuzumab-PS101, pMAZ-IgH-trastuzumab-PS102, pMAZ-IgH-trastuzumab-PS107, pMAZ-IgH-rituximab, pMAZ-IgH-rituximab-VLPLL, pMAZ-IgH-rituximab-DE, pMAZ-IgH-rituximab-PS101, pMAZ-IgH-rituximab-PS102, pMAZ-IgH-rituximab-PS107, pMAZ-cetuximab, pMAZ-IgH-cetuximab-VLPLL, pMAZ-IgH-cetuximab-DE, pMAZ-IgH-cetuximab-PS101, pMAZ-IgH-cetuximab-PS102, or pMAZ-IgH-cetuximab-PS107) was co-transfected with the corresponding IgG light chain plasmid (pMAZ-IgL-GlycoT, pMAZ-IgL-rituximab, or pMAZ-IgL-cetuximab) into Expi293F cells using PEI-Max. After incubation in FreeStyle™ 293 expression medium at 37°C with 8% CO_2 for 7 days, the cells were harvested by centrifugation at 2,000 × g for 10 min, and the collected supernatants were mixed with 1/25 volume of 25× PBS, followed by filteration through a 0.2-μm bottle-top filter. The filtered supernatants were then mixed with 1 ml of Protein A agarose and incubated overnight at 4°C. The resin was transferred to a polypropylene column and washed twice with 5 column volumes (CV) of 1× PBS. Trastuzumab-, rituximab- and cetuximab-Fc variants were eluted using 3 ml of 100 mM glycine-HCl (pH 2.7) and immediately

neutralized by mixing with 1 ml of 1 M Tris (pH 8.0). The buffer was subsequently exchanged to 1× PBS and concentrated using Amicon Ultra-4 spin columns (3-kDa cutoff). For in vivo experiments, the buffer was further exchanged with endotoxin-free DPBS using the NGC chromatography system (Bio-Rad, Hercules, CA, USA).

For the expression and purification of Fc receptors, human, mouse, and cynomolgus Fc receptors (dimeric FcγRIIa-131H-GST, dimeric FcγRIIa-131R-GST, dimeric FcγRIIb-GST, dimeric FcγRIIIa-158V-GST, dimeric FcγRIIIa-158F-GST, dimeric FcRn-GST, dimeric mFcγRIIb-GST, dimeric mFcγRIII-GST, dimeric mFcγRIV-GST, dimeric cFcγRIIa-GST, dimeric cFcγRIIb-GST, dimeric cFcγRIII-GST, monomeric FcγRIIIa-158V-His, monomeric FcγRIIIa-158F-His and monomeric FcγRIIb-His) were expressed and purified as previously described [4, 6]. For the production of dimeric FcγRI-GST, dimeric mFcγRI-GST, and dimeric cFcγRI-GST, Expi293F cells were transfected with plasmids (pMAZ-FcγRI-GST, pMAZ-mFcγRI-GST, or pMAZ-cFcγRI-GST) using PEI-Max and incubated at 30°C with 8% CO_2 for 7 days. For the production of dimeric mFcRn-GST and dimeric cFcRn-GST, plasmids encoding the FcRn α chain and β2m (pMAZ-mFcRn α chain-GST and pMAZ-mβ2m-GST, pMAZ-cFcRn α chain-GST and pMAZ-cβ2m-GST) were co-transfected into Expi293F cells and incubated at 37°C with 8% CO_2 for 7 days. The purification process for these Fc receptors followed the same procedure described in our previous publications [2, 4].

ELISA Assays for Human, Mouse, Cynomolgus Monkey FcγRs Binding

To analyze the binding of antibody-Fc variants to Fcγ receptors (FcγRs), a flat-bottom, high-binding 96-well microplate was coated with 50 μl of dimeric hFcγRs-GST (hFcγRI-GST, hFcγRIIb-GST, hFcγRIIa-131H-GST, hFcγRIIa-131R-GST, hFcγRIIIa-158V-GST, hFcγRIIIa-158F-GST), mFcγRs-GST (mFcγRI-GST, mFcγRIIb-GST, mFcγRIII-GST, mFcγRIV-GST) or cFcγRs-GST (cFcγRI-GST, cFcγRIIa-GST, cFcγRIIb-GST, cFcγRIII-GST) at a concentration of 4 μg/ml after dilution in 0.05 M Na_2CO_3 (pH 9.6), followed by overnight incubation at 4°C. The plate was then blocked with 4% skim milk in PBS and incubated at room temperature for one hour. After four washes with 180 μl of 0.05% PBST (PBS containing 0.05% Tween20), serially diluted trastuzumab-, rituximab-, or cetuximab-Fc variants in 1% skim milk in PBS were added (50 μl per well) and incubated at room temperature for one hour. The plate was then washed again, followed by the addition of 50 μl of HRP-conjugated Protein L (GenScript, Scotch Plains, NJ, USA) and incubation at room temperature for one hour. After further washes, 50 μl of 1-Step Ultra TMB-ELISA substrate solution was added to develop the colorimetric reaction, and the reaction was terminated by adding 50 μl of 2 M H_2SO_4. Absorbance at 450 nm was measured using an Epoch microplate spectrophotometer (BioTek, Winooski, VT, USA).

ELISA Assays for pH-Dependent Human, Mouse, Cynomolgus Monkey FcRn Binding

To evaluate the pH-dependent FcRn binding of antibody-Fc variants, trastuzumab-, rituximab-, or cetuximab-Fc variants were coated onto microplates at a concentration of 4 μg/ml in 0.05 M Na_2CO_3 (pH 9.6), and incubated at 4°C for 16 hours. The plate was then

blocked with 4% skim milk in PBS and incubated at room temperature for one hour, followed by four washes with PBS. For the analysis of hFcRn binding at neutral and weakly acidic pH conditions, 0.05% PBST at pH 7.4 (neutral) and pH 6.0 (weakly acidic) were used. Fifty microliters (50 μl) of serially diluted hFcRn-GST, mFcRn-GST, or cFcRn-GST in 1% skim milk in PBS (pH 7.4) or PBS (pH 6.0), were added to each well and incubated at room temperature for one hour. After washing, 50 μl of goat anti-GST-HRP conjugate was added and incubated at room temperature for one hour. Color development and absorbance measurement followed the procedure described above.

CHO-hFcγRIIIa and -cFcγRIII Binding Analysis

To simulate the interaction between FcγRIIIa expressed on NK cell surfaces and trastuzumab-Fc variants, CHO cells stably expressing hFcγRIIIa-158V, hFcγRIIIa-158F, or cFcγRIII were generated. The genes encoding hFcγRIIIa-158V, hFcγRIIIa-158F, and cFcγRIII were cloned into the pcDNA5-FRT plasmid. The constructed plasmids (pcDNA5-hFcγRIIIa-158V, pcDNA5-hFcγRIIIa-158F and pcDNA5-cFcγRIII) were co-transfected with pOG44-Flp into Flp-in CHO cells^{TM} at a 1:9 ratio to induce site-specific gene recombination. To select CHO cells with successful gene integration, a hygromycin-B resistance gene was incorporated into the plasmid. Following transfection, cells were cultured in complete growth medium (Ham's F12 Nutrient Mix, GlutaMAX supplemented with 10% of FBS) containing 500 μg/ml hygromycin-B. As a result, stable CHO cell lines expressing hFcγRIIIa-158V, hFcγRIIIa-158F, or cFcγRIII were successfully established (CHO-FcγRIIIa-158V, CHO-

FcγRIIIa-158F, and CHO-cFcγRIII). For binding analysis, 4 × 10^6 of the established CHO-FcγRIIIa-158V, CHO-FcγRIIIa-158F, and CHO-cFcγRIII cells were incubated with 2.5 nM trastuzumab Fc variants and 2.5 nM HER2-AF488 fluorescent probes on ice for 30 min. The incubated cells were then washed twice with cold PBS, and analyzed by flow cytometry (FACSLyric™).

HER2 Binding Analysis Using SK-BR-3

To evaluate whether the binding affinity to HER2 was preserved following the introduction of Fc variants into the trastuzumab model antibody, a cell-based antigen binding assay was performed using the HER2-expressing SK-BR-3 cell line. A total of 4 × 10^6 SK-BR-3 cells were incubated with 10 nM of trastuzumab-Fc variants and Protein A-FITC on ice for 30 min. After two washes with cold PBS, samples were analyzed by flow cytometry (FACSLyric™, BD, Franklin Lakes, NJ, USA) to assess binding via FITC fluorescence intensity.

Genotyping of FcγRIIa and FcγRIIIa

To determine the FcγRIIIa-158V/F genotype of the effector cells, 200 µl of peripheral blood was collected from each donor prior to immune cell isolation. Genomic DNA was extracted using the AccuPrep® Plant Genomic DNA Extraction Kit (Bioneer, Daejeon, Republic of Korea). To genotype *FCGR2A*, genomic DNA was PCR-amplified using primers MJ#629 and MJ#630 to target the single nucleotide polymorphism (SNP) at position 131

(His/Arg), followed by Sanger sequencing for allelic determination. A single peak corresponding to A (CAT) or G (CGT) indicated homozygosity (131His/His or 131Arg/Arg, respectively), whereas the presence of both A and G peaks indicated heterozygosity (131His/Arg). For FcγRIIIa genotyping, the SNP at position 158 of the *FCGR3A* gene, which encodes either valine (Val) or phenylalanine (Phe), was amplified by PCR using e-Taq polymerase with a mixture of primers MJ#625–628. PCR conditions consisted of an initial denaturation at 96°C for 2 min, followed by 10 cycles of 95°C for 30 sec, 70°C for 30 sec, and 72°C for 30 sec, and then 17 cycles of 96°C for 30 sec, 68°C for 30 sec, and 72°C for 30 sec. A final extension was performed at 72°C for 5 min [7]. PCR products were analyzed by electrophoresis on a 2% agarose gel, and donor genotypes were determined as 158Val/Val, 158Val/Phe, or 158Phe/Phe, based on banding patterns.

Analysis of ADCP Activity for Trastuzumab-Fc Variants

PBMCs were freshly isolated from healthy donor blood using density gradient centrifugation with Histopaque-1.077 (Sigma-Aldrich). The PBMC layer was washed twice with cold PBS to remove platelets and subsequently used for monocyte isolation. To generate monocyte-derived macrophages, $CD14^+$ monocytes were isolated using CD14 MicroBeads (Miltenyi Biotec, Bergisch Gladbach, Germany) and cultured in RPMI 1640 medium supplemented with 15% FBS and 50 ng/ml GM-CSF for one week to allow differentiation into macrophages. For phagocytic activity analysis, 4×10^4 SK-BR-3 cells labeled with PKH67 Green Fluorescent Cell Linker were incubated with 500 ng/ml

antibodies, 10% human serum and 2×10^5 macrophages at an E:T ratio of 5:1 in a 96-well V-bottom plate for 4 hours. After incubation, the cells were harvested using Accutase (Innovative Cell Technologies, San Diego, CA, USA), and macrophages were stained with APC-conjugated anti-CD11b and anti-CD14 antibodies. Phagocytic activity was assessed using a BD FACSLyric^{TM} flow cytometer and calculated as the percentage of macrophages double-positive for PKH67 and CD11b/CD14 relative to the total tumor cell population.

Analysis of Pharmacokinetic Profile

To assess the pharmacokinetic profiles of trastuzumab-Fc variants (trastuzumab, trastuzumab-VLPLL, trastuzumab-DE, trastuzumab-PS101, and trastuzumab-PS107) in hFcRn transgenic (Tg) mice (B6.Cg-*Fcgrt*^{tm1Dcr} Tg(CAG-FCGRT)276Dcr/DcrJ hemizygous), trastuzumab-Fc variants were administered via intravenous injection at a dose of 5 mg/kg (n = 3). Blood samples were obtained from the facial vein at predetermined time points post-injection: 0.5, 24, 168, 336, 504, 672, 840, 1,008, and 1,176 hours. To quantify the serum concentration of trastuzumab-Fc variants, 96-well microplates were coated with 4 µg/ml HER2 diluted in 0.05 M Na_2CO_3 (pH 9.6) and incubated overnight at 4°C. The plates were then blocked with 4% skim milk in PBS at room temperature for one hour, followed by four washes with 180 µl of 0.05% PBST. Serially diluted mouse serum samples and trastuzumab-Fc variants (used as a standard) were added to the plates and incubated at room temperature for one hour. After washing four times with 0.05% PBST, 50 µl of HRP-conjugated AffiniPure F(ab')$_2$ fragment goat anti-human IgG (H+L) was added and

incubated at room temperature for one hour. Following additional washes, 50 µl of 1-Step Ultra TMB substrate solution was added to develop the colorimetric signal, and the reaction was quenched by adding 50 µl of 2 M H_2SO_4. Absorbance was measured at 450 nm using an Epoch microplate spectrophotometer (BioTek).

Prediction of Fc–hFcγRIIIa Structure and Immunogenicity

Structural prediction of Fc–hFcγRIIIa complexes was conducted using the AlphaFold3 server, which generates high-confidence models of protein-protein interactions. The resulting coordinate files were downloaded in CIF format and converted to standard PDB files using PyMOL v2.x (Schrödinger, LLC) for visualization. Interface analyses of Fc–hFcγRIIIa interactions were carried out using LigPlot+ V.2.2.9, (EMBL-EBI, Cambridgeshire, UK), which delineates hydrogen bonding networks and hydrophobic contacts within the complex. Structural comparison and interpretative visualization were further performed using PyMOL. To evaluate the potential immunogenicity of Fc variants, in silico T cell epitope profiling was performed via the Immune Epitope Database (IEDB, v2.22) analysis resource. Linear 15-mer peptides derived from each Fc variant (wild-type, VLPLL, DE, PS101, PS102, PS107) were computationally assessed for predicted binding affinity a reference panel of 27 high-frequency human MHC class II (HLA) alleles [2]. Predicted affinities were reported as percentile rank scores, with lower percentile values indicating stronger MHC binding. The compiled output was visualized as heatmaps to facilitate comparative immunogenicity profiling.

Supplementary Figures

Supplementary Fig. S1 Relative binding of trastuzumab-Fc variants to FcγRIIIa and FcγRIIb. **(A–B)** Fc binding profiles of trastuzumab-Fc variants were evaluated by comparing their relative binding signals to FcγRIIIa-158V **(A)** and FcγRIIIa-158F **(B)** versus FcγRIIb. Relative binding signals were measured by ELISA.

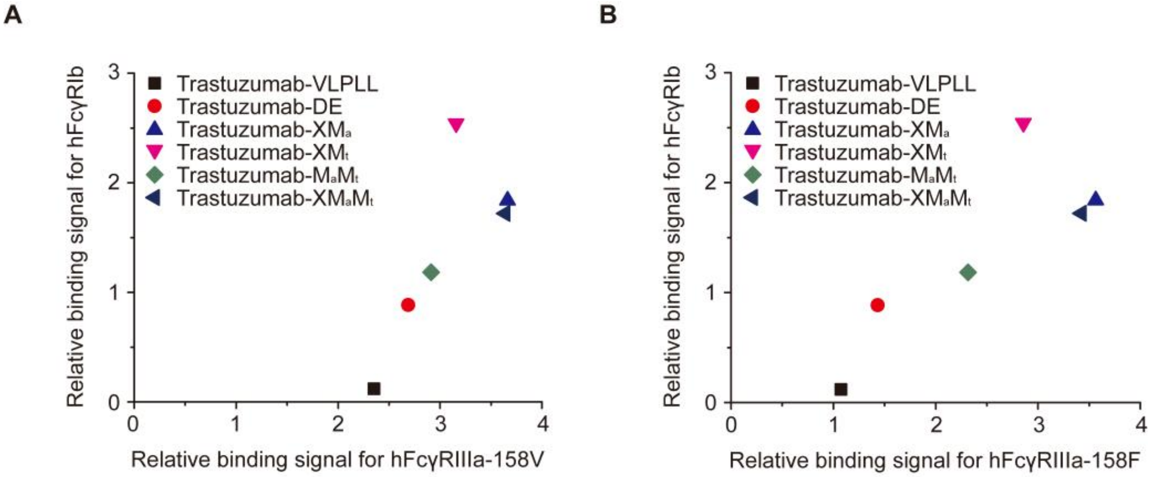

- XM_a: S239D/F243L/R292P/Y300L/V305I/A330L/I332E/P396L
- XM_t: S239D/P247I/A330L/I332E/A339Q
- M_aM_t: F243L/P247I/R292P/Y300L/V305I/A339Q/P396L
- XM_aM_t: S239D/F243L/P247I/R292P/Y300L/V305I/A330L/I332E/A339Q/P396L

Supplementary Fig. 2 Diagram of FRT site specific Flp recombination mechanism. pcDNA5-FRT expression vector containing Fc-transmembrane of PDGFR (PDGFR TM) was integrated to the genomic DNA of Flp-in CHO cells by co-transfection with Flp recombinase encoding plasmid (pOG44-Flp).

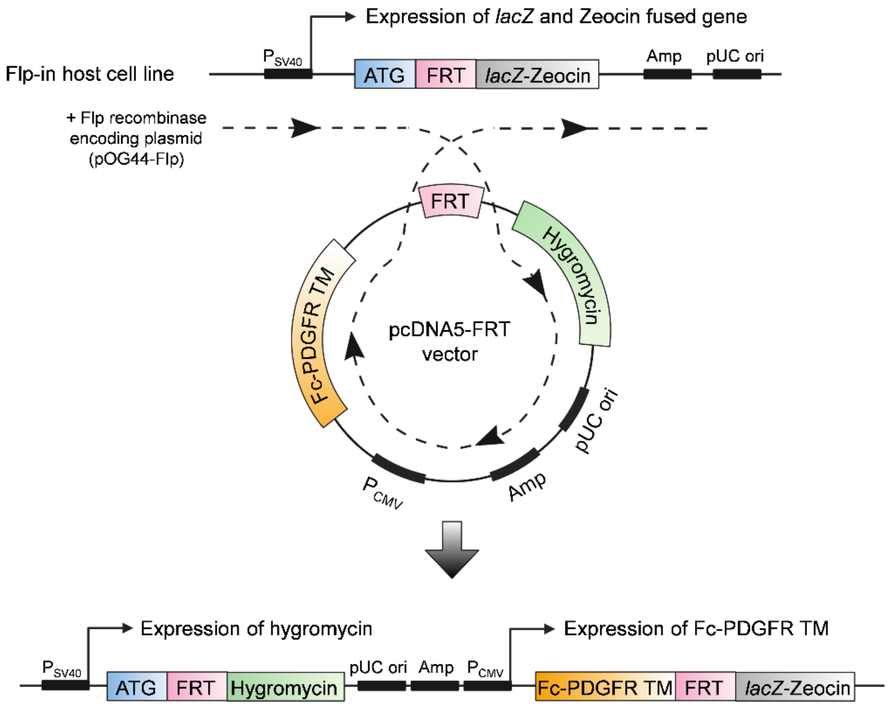

Supplementary Fig. 3 Screening and identification of glycosylated Fc variants. **(A)** Gating for glycosylated Fc variants library sorting using AriaIII. **(B)** Sequence alignment of wild-type Fc and Fc variants.

A

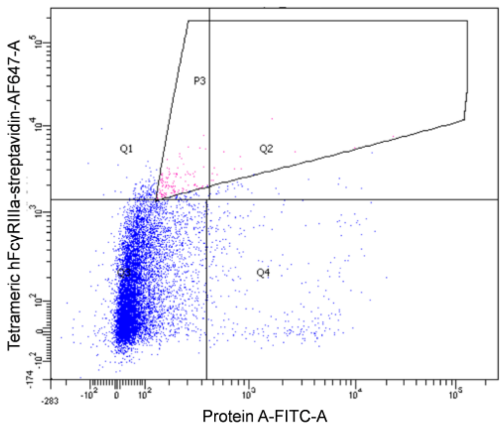

B

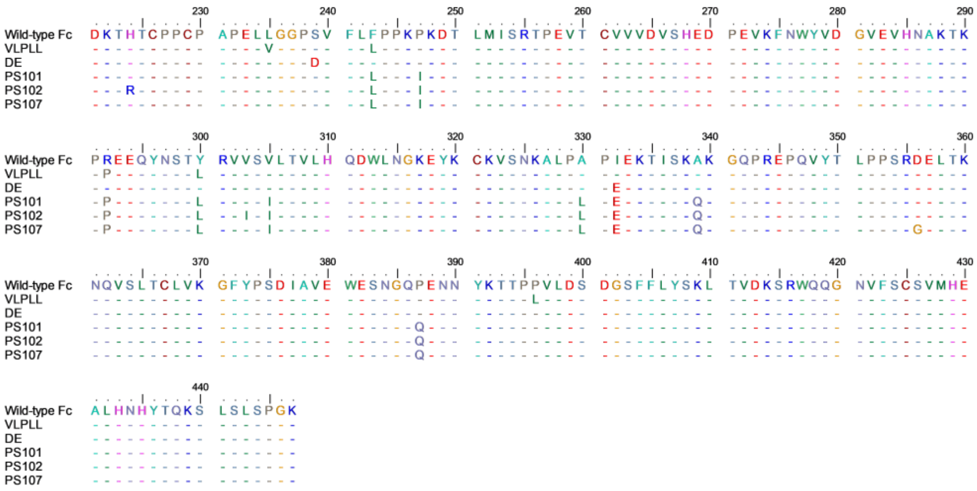

Supplementary Fig. 4 Binding kinetics of trastuzumab and trastuzumab-Fc variants to FcγRIIIa-158V, FcγRIIIa-158F and FcγRIIb measured by Octet R8 instrument. Anti-human Fab-CH1 biosensors (FAB2G) were loaded with 500 nM of each trastuzumab-Fc variant for 300 sec. After a 60 sec baseline in PBS, the biosensors were transferred to wells containing monomeric His-tagged FcγRIIIa-158V, FcγRIIIa-158F, or FcγRIIb diluted in PBS for a 30 sec association phase, followed by a 300 sec dissociation phase in PBS. For steady-state affinity measurements, the dissociation step was shortened to 30 sec. Sensorgrams represent the binding responses at various receptor concentrations, and steady-state binding levels (insets) were used to calculate the apparent equilibrium dissociation constants.

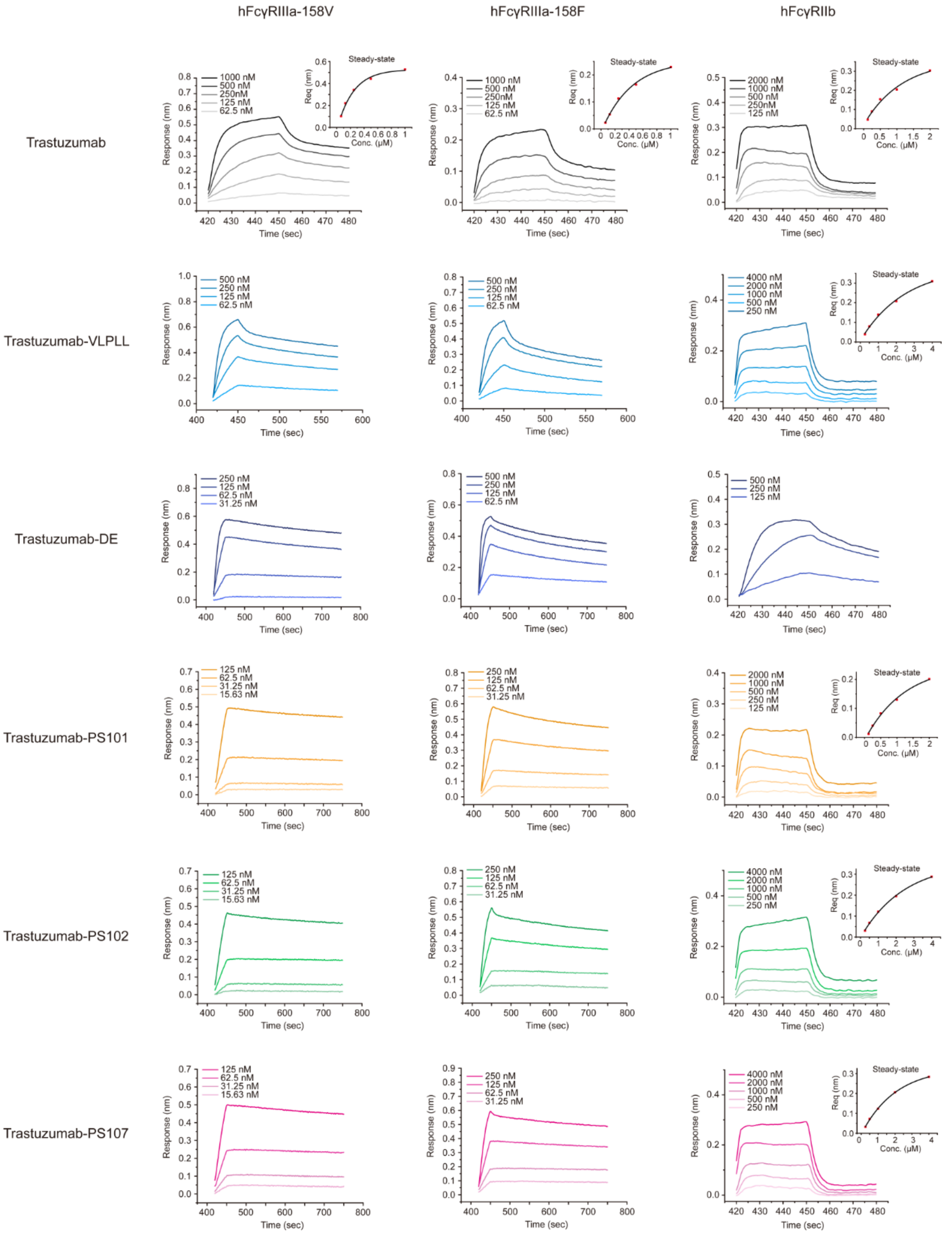
hFcγRIIIa-158V
hFcγRIIIa-158F
hFcγRIIb
Trastuzumab
Trastuzumab-VLPLL
Trastuzumab-DE
Trastuzumab-PS101
Trastuzumab-PS102
Trastuzumab-PS107
Steady-state
Req (nm)
Conc. (μM)
Response (nm)
Time (sec)
2000 nM
1000 nM
500 nM
250nM
250 nM
125 nM
62.5 nM
31.25 nM
15.63 nM
4000 nM

Supplementary Fig. 5 Preparation and binding characterization of trastuzumab and trastuzumab-Fc variants. **(A)** SDS-PAGE of trastuzumab and Fc-engineered variants (-VLPLL, -DE, -PS101, -PS102 and -PS107) produced in Expi293F cells and purified by protein A affinity chromatography. **(B–F)** ELISA assays of trastuzumab and trastuzumab-Fc variants. Binding to hFcγRI **(B)**, hFcγRIIa-131H **(C)**, hFcγRIIa-131R **(D)**, and hFcRn at pH 6.0 **(E)** and pH 7.4 **(F)** were measured. Data represent mean ± SD from duplicate measurements.

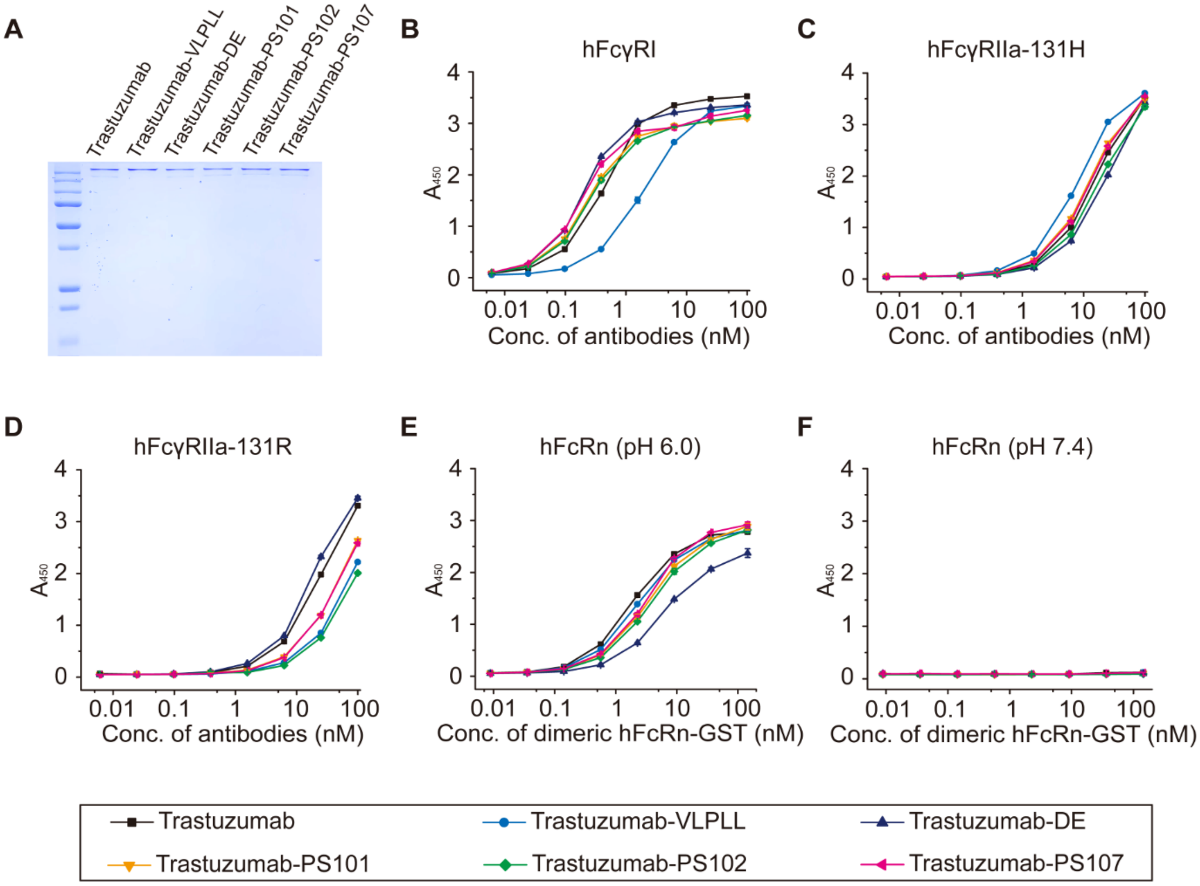

Supplementary Fig. 6 Antigen binding analysis of trastuzumab and trastuzumab-Fc variants to HER2-expressing cells by flow cytometry. SK-BR-3 cells, which express high levels of HER2, were incubated with 10 nM of trastuzumab or Fc-engineered variants (-VLPLL, -DE, -PS101, -PS102, -PS107), followed by staining with FITC-conjugated protein A for antibody detection. The first histogram (gray) represents the negative control sample treated with Protein A-FITC only (no antibody).

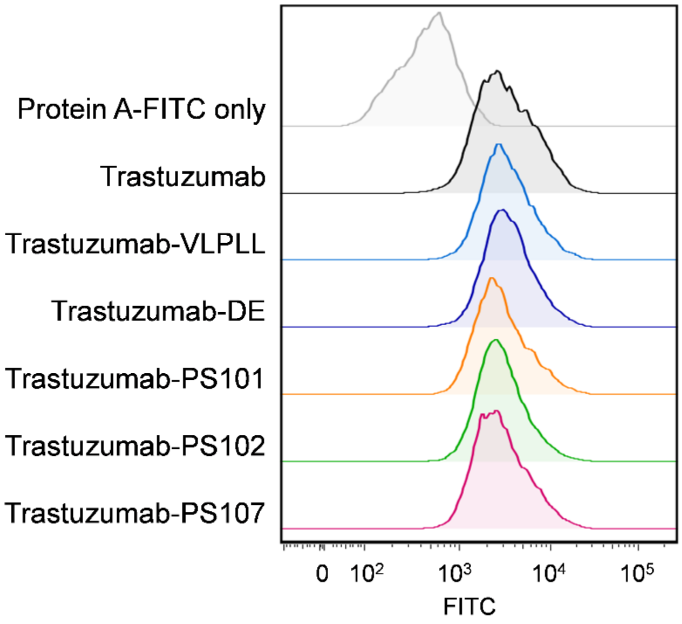

Supplementary Fig. 7 Comparative analysis of HER2 surface expression among various cell lines using trastuzumab-based flow cytometry. **(A–E)** Cell surface HER2 expression was assessed in CHO **(A)**, MDA-MB-231 **(B)**, JIMT-1 **(C)**, MDA-MB-453 **(D)**, and SK-BR-3 **(E)** cell lines by FACS. Cells were stained with either protein A-FITC only (gray histogram, negative control) or with 50 nM trastuzumab followed by protein A-FITC (green or yellow histograms). **(F)** Quantification of HER2 expression based on normalized median fluorescence intensity (MFI), calculated by dividing the MFI of trastuzumab-stained cells by that of the corresponding protein A-FITC only control.

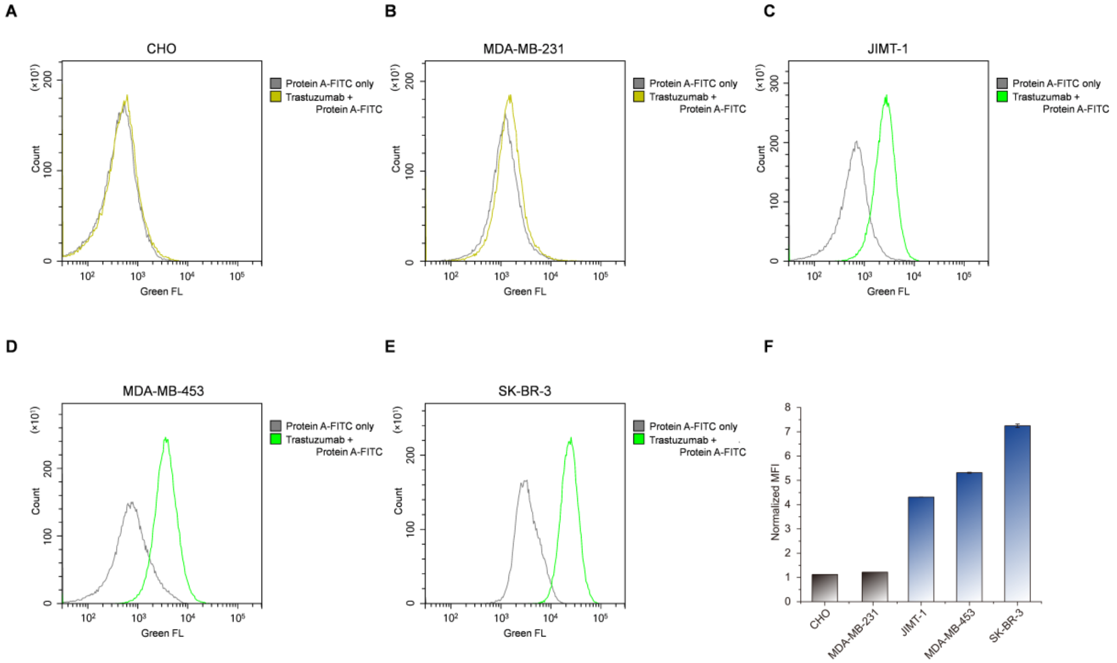

Supplementary Fig. 8 KT50 and KT20 values of trastuzumab and trastuzumab-Fc variants against various HER2-positive target cells. Cytolysis was assessed using the xCELLigence real-time cell analysis system with hPBMCs as effector cells. **(A)** SK-BR-3 cells were used as target cells at an E:T ratio of 5:1, and concentration of antibodies were 100 pM. **(B)** MDA-MB-453 cells were used as target cells with an E:T ratio of 2:1, and concentration of antibodies were 20 pM. **(C)** JIMT-1 cells were used as target cells at an E:T ratio of 10:1, and concentration of antibodies were 20 pM (n.d., non-determined).

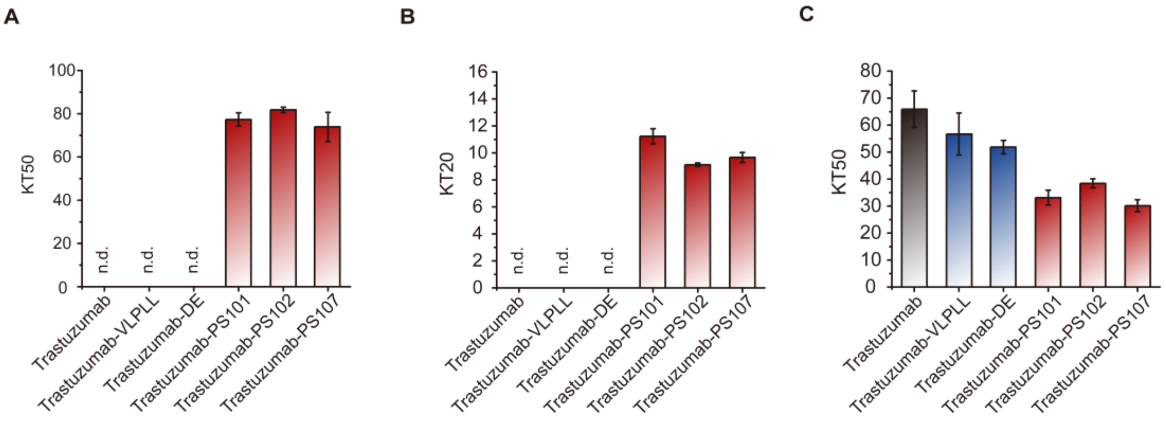

	KT50 (h)
Trastuzumab	n.d.
Trastuzumab-VLPLL	n.d.
Trastuzumab-DE	n.d.
Trastuzumab-PS101	77.3±3.09
Trastuzumab-PS102	81.8±1.28
Trastuzumab-PS107	73.9±6.76

	KT20 (h)
Trastuzumab	n.d.
Trastuzumab-VLPLL	n.d.
Trastuzumab-DE	n.d.
Trastuzumab-PS101	11.2±0.56
Trastuzumab-PS102	9.1±0.12
Trastuzumab-PS107	9.7±0.36

	KT50 (h)
Trastuzumab	65.9±6.77
Trastuzumab-VLPLL	56.7±7.79
Trastuzumab-DE	51.9±2.48
Trastuzumab-PS101	33.1±2.76
Trastuzumab-PS102	38.4±1.66
Trastuzumab-PS107	30.1±2.22

Supplementary Fig. 9 ELISA assays of cetuximab and cetuximab-Fc variants. Binding to hFcγRI **(A)**, hFcγRIIa-131H **(B)**, hFcγRIIa-131R **(C)** and hFcRn at pH 6.0 **(D)** and pH 7.4 **(E)** were measured. Data represent mean ± SD from duplicate measurements.

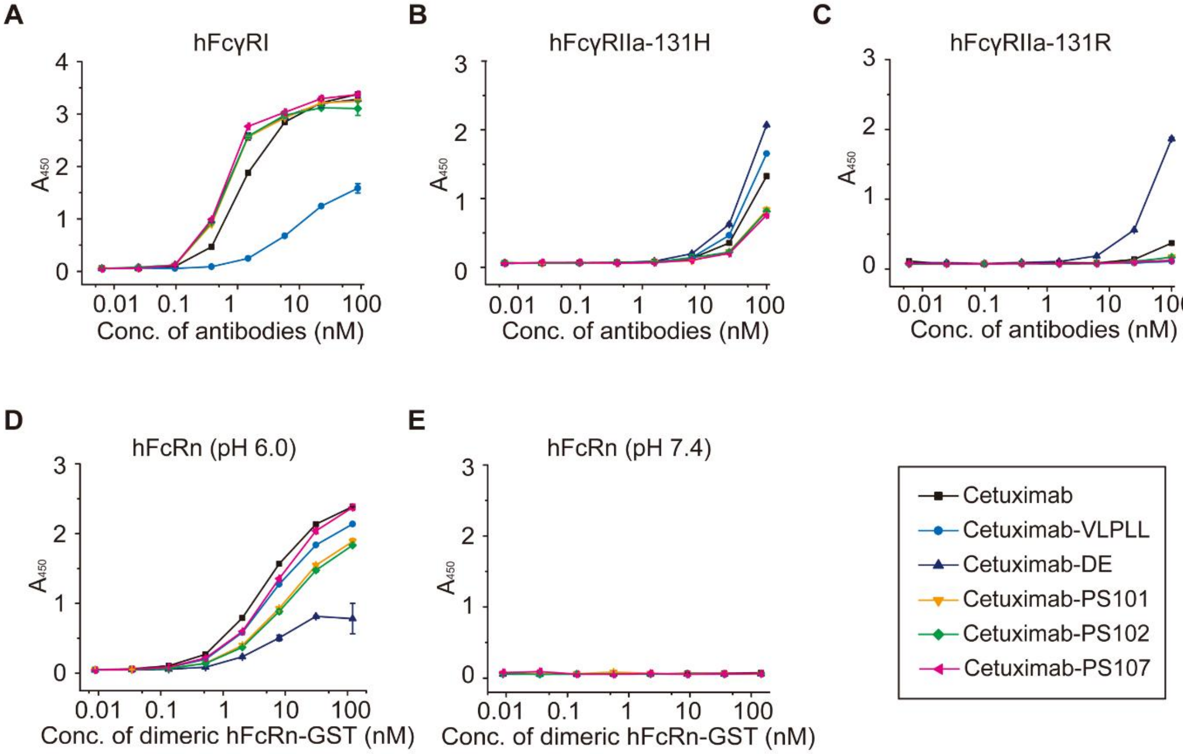

Supplementary Fig. 10 Cytolytic activity of cetuximab and Fc-engineered variants against EGFR-positive A431 cell. **(A)** Antibodies were used at a final concentration of 20 pM, and E:T ratio was set at 5:1. Endpoint cytolysis was quantified at 33 h post-incubation. **(B)** Antibodies were used at 100 pM with an E:T ratio of 2:1, and endpoint cytolysis was measured at 35 h. Data represent mean ± SD from triplicate measurements. Statistical significance was determined using two-tailed unpaired student's t-test. ns, not significant ($p > 0.05$).

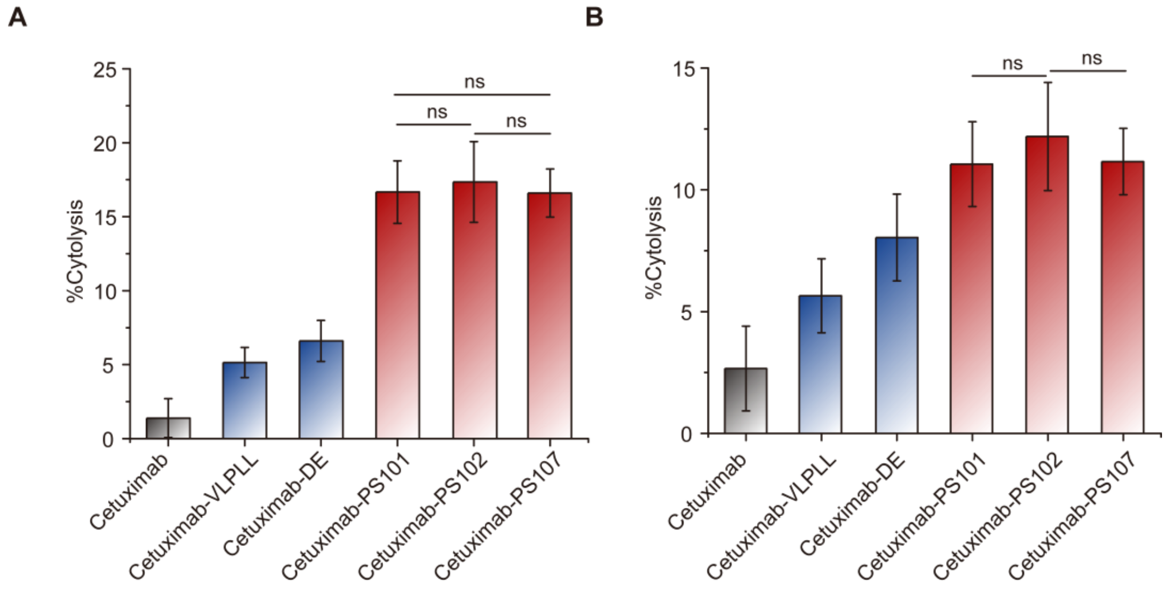

Supplementary Fig. 11 ELISA assays of rituximab and rituximab-Fc variants. Binding to hFcγRI **(A)**, hFcγRIIa-131H **(B)**, hFcγRIIa-131R **(C)** and hFcRn at pH 6.0 **(D)** and pH 7.4 **(E)** were measured. Data represent mean ± SD from duplicate measurements.

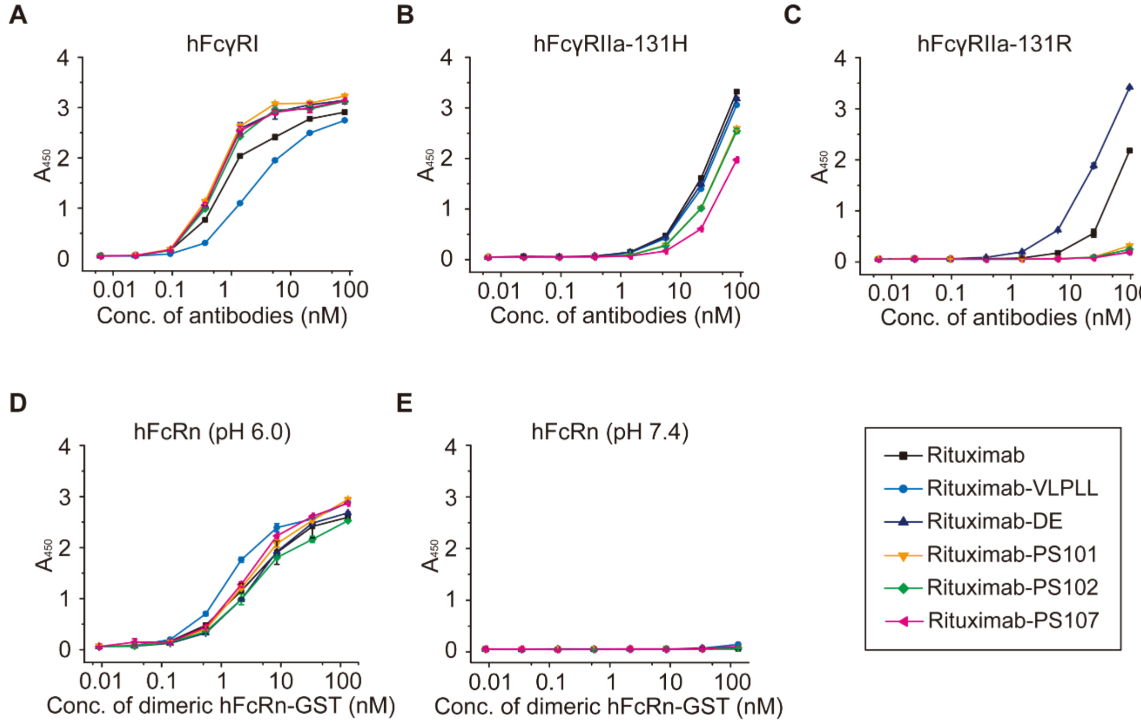

Supplementary Fig. 12 Expression and purification of mouse and cynomolgus monkey Fc receptors. **(A–B)** SDS-PAGE of recombinant Fc receptors from mouse (mFcγRI, mFcγRIIb, mFcγRIII, mFcγRIV, and mFcRn) **(A)** and cynomolgus monkey (cFcγRI, cFcγRIIa, cFcγRIIb, cFcγRIII, and cFcRn) **(B)**, produced in Expi293F cells and purified by glutathione affinity chromatography.

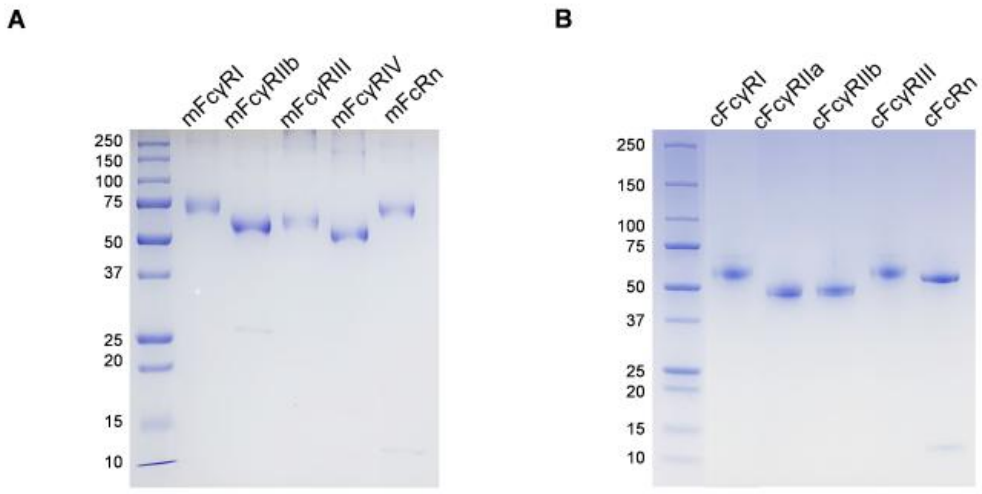

Supplementary Fig. 13 Binding profiles of trastuzumab and engineered Fc variants to mFcRn analyzed by ELISA. **(A)** Binding was assessed under mildly acidic conditions (pH 6.0), mimicking the endosomal environment. **(B)** Binding was measured under neutral conditions (pH 7.4), mimicking the physiological extracellular environment. Error bars represent the SD from duplicate measurements.

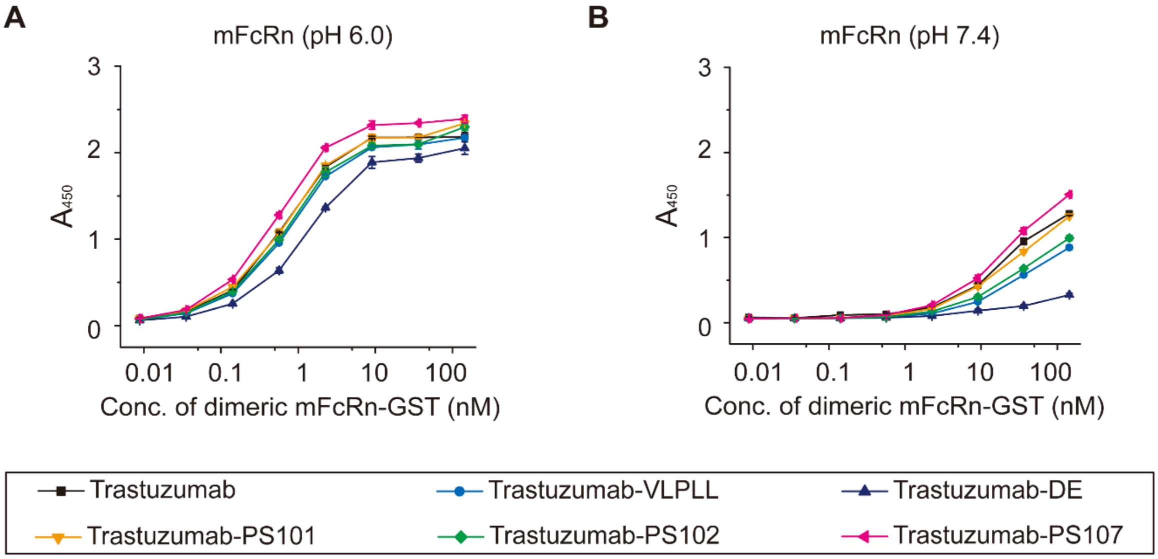

Supplementary Fig. 14 Binding profiles of trastuzumab and engineered Fc variants to cFcRn analyzed by ELISA. **(A)** Binding was assessed under mildly acidic conditions (pH 6.0). **(B)** Binding was measured under neutral conditions (pH 7.4). Error bars represent the SD from duplicate measurements.

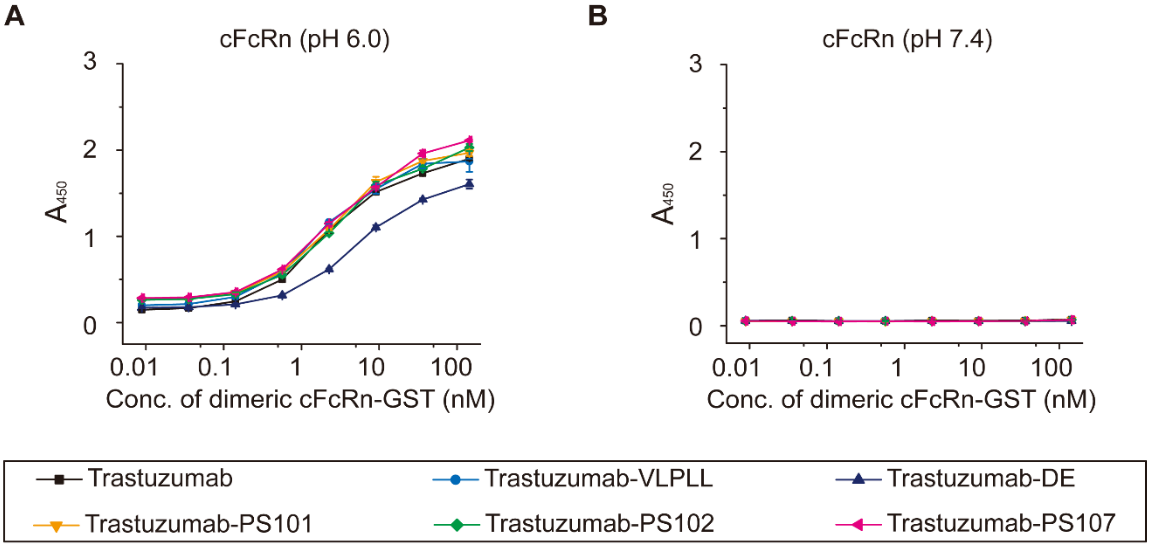

Supplementary Fig. 15 Binding profiles of cetuximab and engineered Fc variants to mouse Fc receptors by ELISA. Binding to mFcγRI **(A)**, mFcγRIIb **(B)**, mFcγRIII **(C)**, mFcγRIV **(D)**, mFcRn at pH 6.0 **(E)** and mFcRn at pH 7.4 **(F)** were measured. Data represent mean ± SD from duplicate measurements.

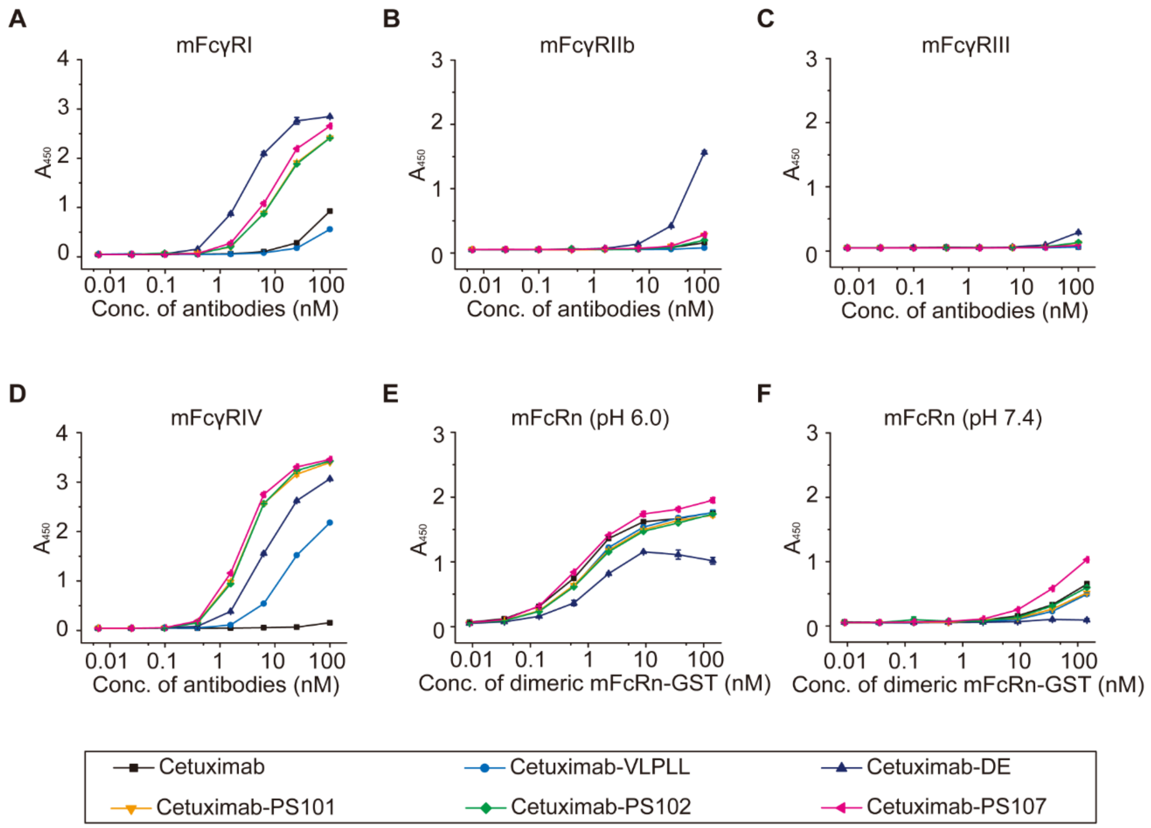

Supplementary Fig. 16 ELISA assays of cetuximab and cetuximab-Fc variants. Binding to cFcγRI **(A)**, cFcγRIIa **(B)**, cFcγRIIb **(C)**, cFcγRIII **(D)**, cFcRn at pH 6.0 **(E)** and cFcRn at pH 7.4 **(F)** were measured. Data represent mean ± SD from duplicate measurements.

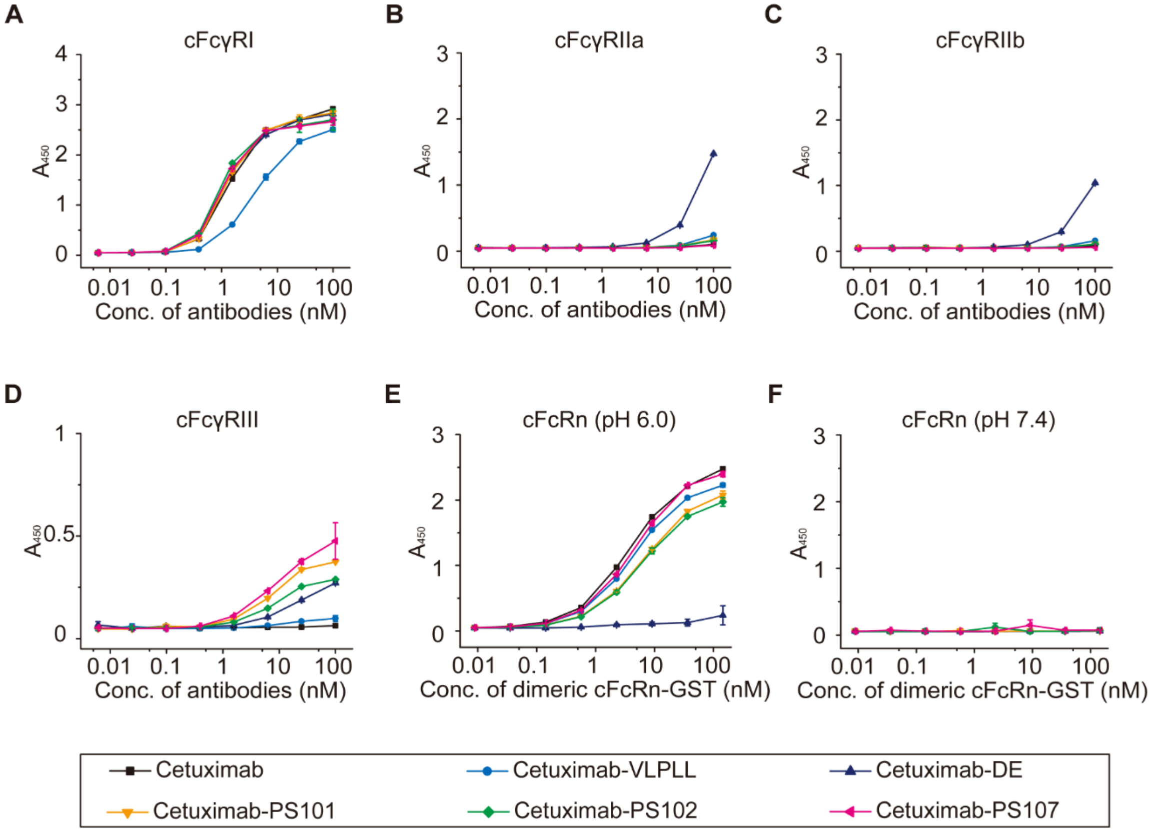

Supplementary Fig. 17 Binding profiles of rituximab and engineered Fc variants to mouse Fc receptors by ELISA. Binding to mFcγRI **(A)**, mFcγRIIb **(B)**, mFcγRIII **(C)**, mFcγRIV **(D)**, mFcRn at pH 6.0 **(E)** and mFcRn at pH 7.4 **(F)** were measured. Data represent mean ± SD from duplicate measurements.

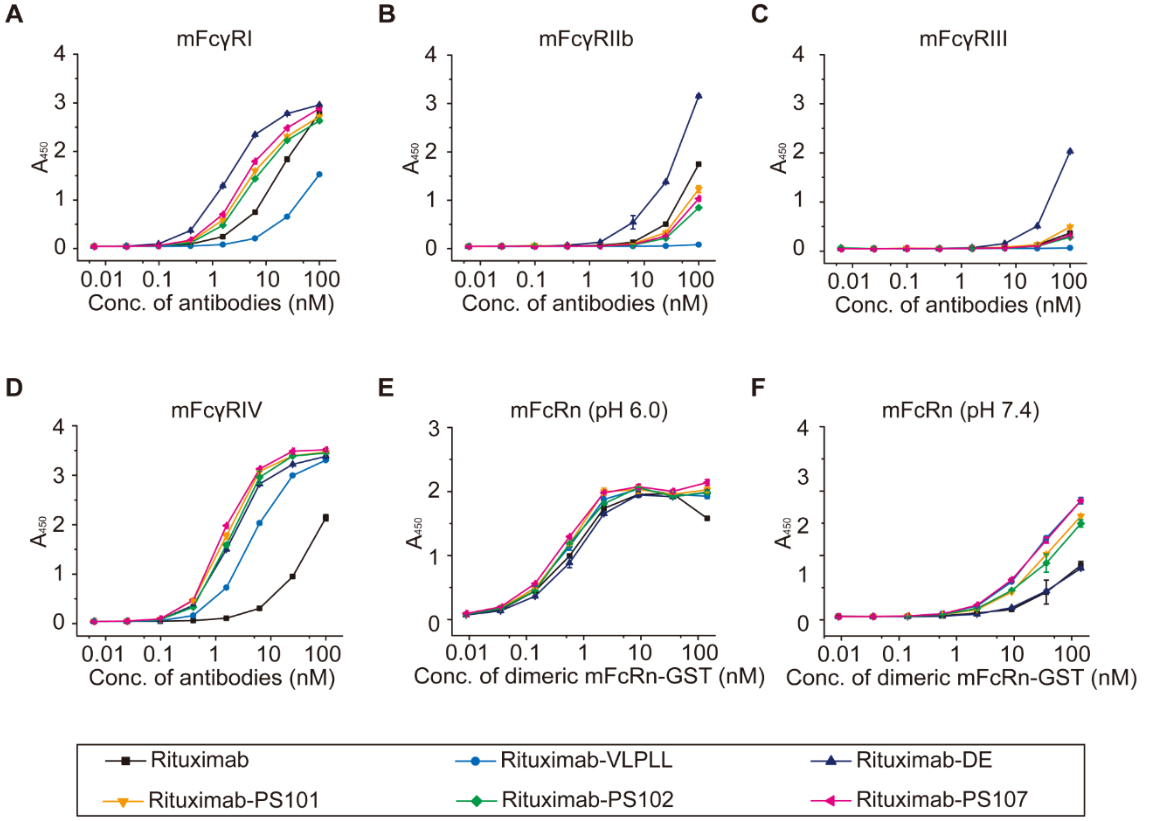

Supplementary Fig. 18 ELISA assays of rituximab and rituximab-Fc variants. Binding to cFcγRI **(A)**, cFcγRIIa **(B)**, cFcγRIIb **(C)**, cFcγRIII **(D)**, cFcRn at pH 6.0 **(E)** and cFcRn at pH 7.4 **(F)** were measured. Data represent mean ± SD from duplicate measurements.

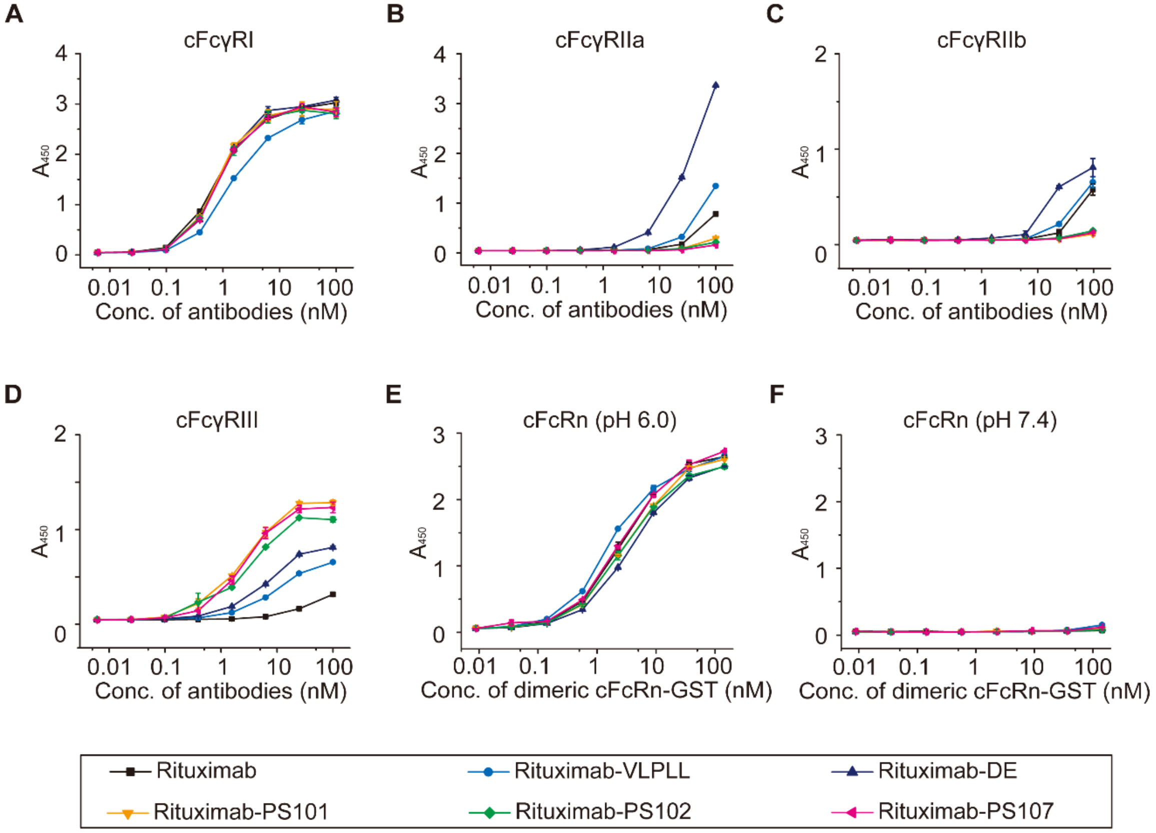

Supplementary Fig. 19 Pharmacokinetic analysis of trastuzumab and trastuzumab-Fc variants in hFcRn Tg mice (Tg32). Serum concentrations of trastuzumab **(A)** and trastuzumab-Fc variants, including trastuzumab-VLPLL **(B)**, trastuzumab-DE **(C)**, trastuzumab-PS101 **(D)** and trastuzumab-PS107 **(E)** were measured following intravenous administration at 5 mg/kg. Error bars represent standard deviations.

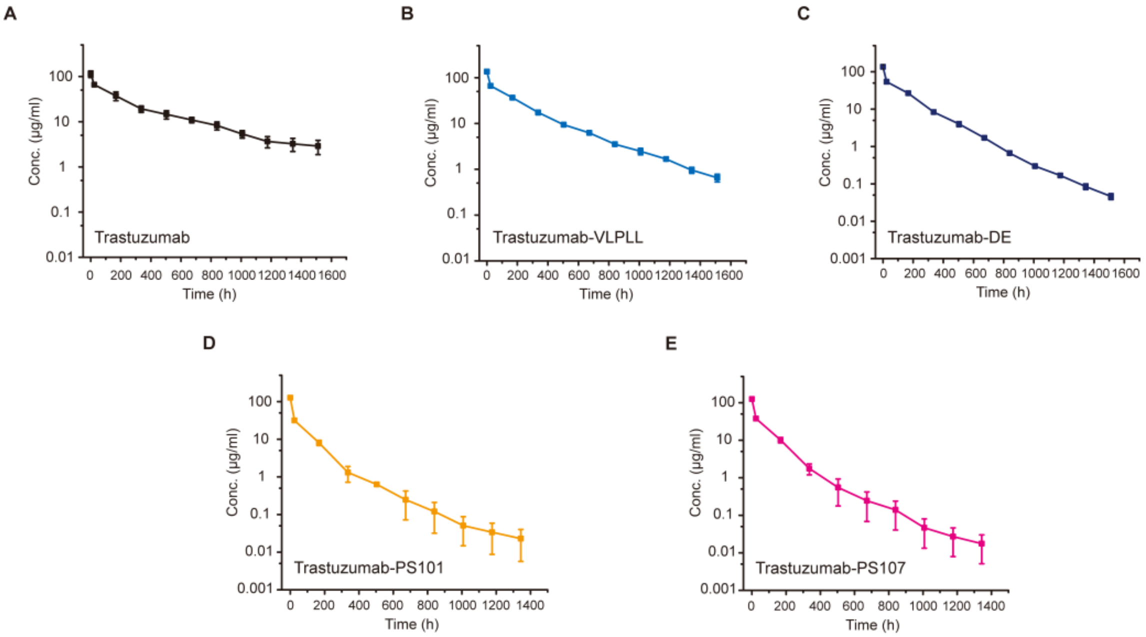

Supplementary Fig. 20 Pharmacokinetic analysis of trastuzumab and trastuzumab-Fc variants in NSG hFcRn Tg mice (NSG Tg32). Serum concentrations of trastuzumab **(A)**, trastuzumab-DE **(B)**, trastuzumab-PS101 **(C)** and trastuzumab-PS107 **(D)** were measured following intravenous administration at 5 mg/kg. Error bars represent standard deviations.

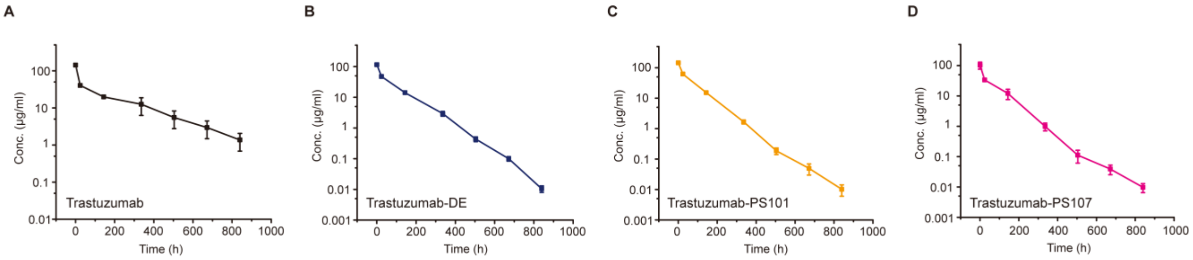

Supplementary Fig. 21 In vivo antitumor efficacy of trastuzumab and Fc-engineered variants in a JIMT-1 xenograft mouse model. Five-week-old female BALB/c nude (CAnN.Cg-Foxn1 nu/CrlOri) mice (*n = 10* per group) were subcutaneously implanted with 5×10^6 JIMT-1 cells in 100 µl of Opti-MEM. When tumor volumes reached approximately 80 mm^3, mice were treated intraperitoneally with 10 mg/kg of each antibody twice per week for a total of six doses (indicated by red arrows). **(A–F)** Individual tumor growth curves for each treatment group: vehicle **(A)**, trastuzumab **(B)**, trastuzumab-VLPLL **(C)**, trastuzumab-DE **(D)**, trastuzumab-PS101 **(E)**, and trastuzumab-PS107 **(F)**. **(G)** Representative images of excised tumors collected on day 46 post-implantation. **(H)** Mouse body weight monitored throughout the study to assess systemic toxicity. Tumor volumes were measured three times per week using calipers and calculated as $(L \times W^2)/2$. Data represent mean ± SD. Statistical comparisons of tumor volumes were performed using one-way ANOVA.

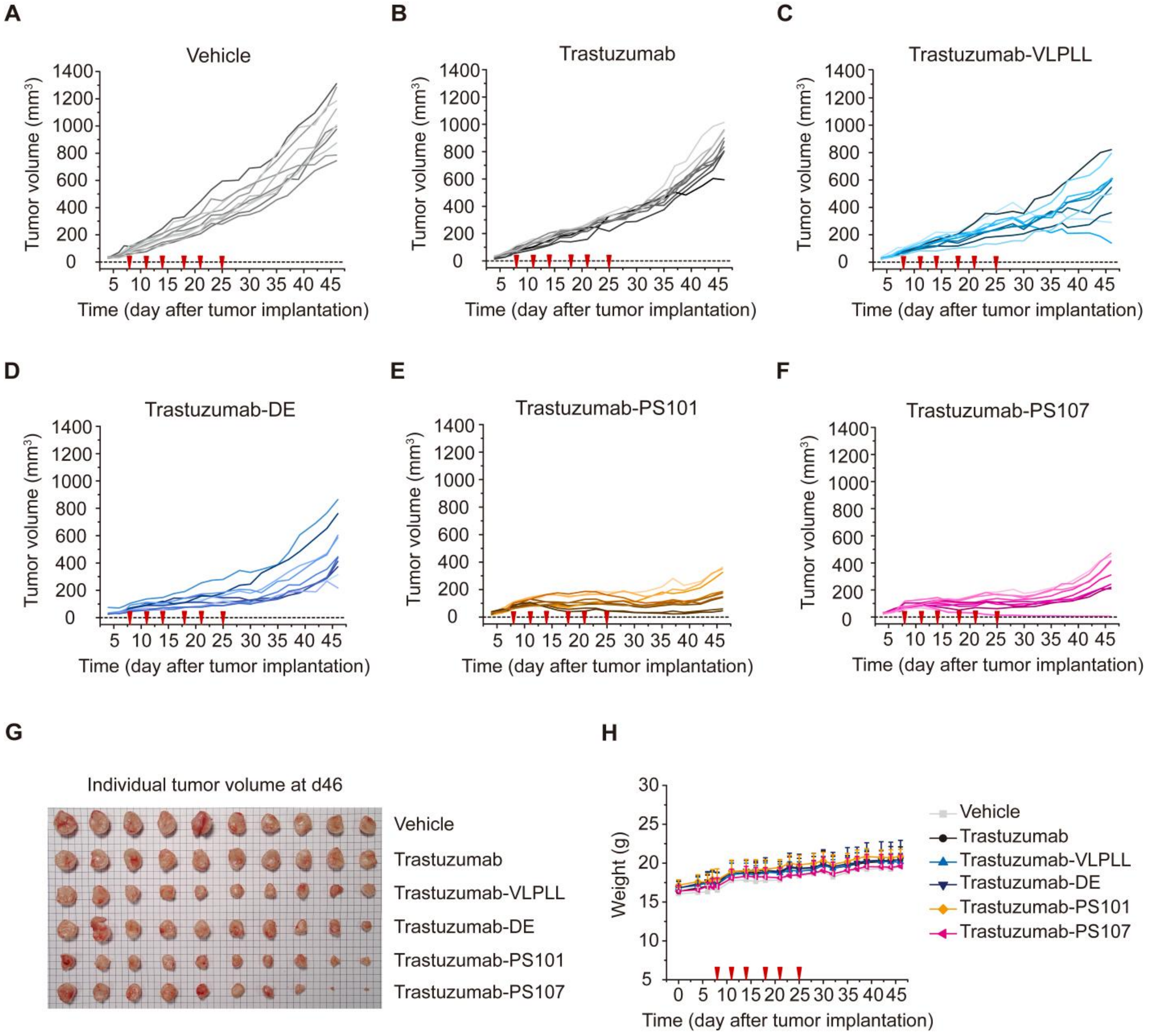
A
Vehicle
B
Trastuzumab
C
Trastuzumab-VLPLL
D
Trastuzumab-DE
E
Trastuzumab-PS101
F
Trastuzumab-PS107
Tumor volume (mm³)
Time (day after tumor implantation)
G
Individual tumor volume at d46
Vehicle
Trastuzumab
Trastuzumab-VLPLL
Trastuzumab-DE
Trastuzumab-PS101
Trastuzumab-PS107
H
Weight (g)
Time (day after tumor implantation)
Vehicle
Trastuzumab
Trastuzumab-VLPLL
Trastuzumab-DE
Trastuzumab-PS101
Trastuzumab-PS107

Supplementary Fig. 22 Structural modeling and interaction analysis of Fc–FcγRIIIa complexes. Representative structural models of Fc–FcγRIIIa complexes were predicted using AlphaFold3, and molecular interactions at the interface were analyzed using LigPlot+ (DIMPLOT) and visualized in PyMOL. **(A–D)** Each panel shows a 3D structure (left) and corresponding 2D schematic diagram (right) indicates the binding interface between Fc and FcγRIIIa are indicated. Interaction between wild-type Fc (B-chain) and FcγRIIIa-158V **(A)**. Interaction between PS107 (B-chain) and FcγRIIIa-158V **(B)**. Interaction between wild-type Fc (B-chain) and FcγRIIIa-158F **(C)**. Interaction between PS107 (B-chain) and FcγRIIIa-158F **(D)**. Key contact residues, including mutation sites (Glu332 and Leu330), are labeled. Mutated residues are colored in red, and receptor chains are shown in blue (158V) or orange (158F).

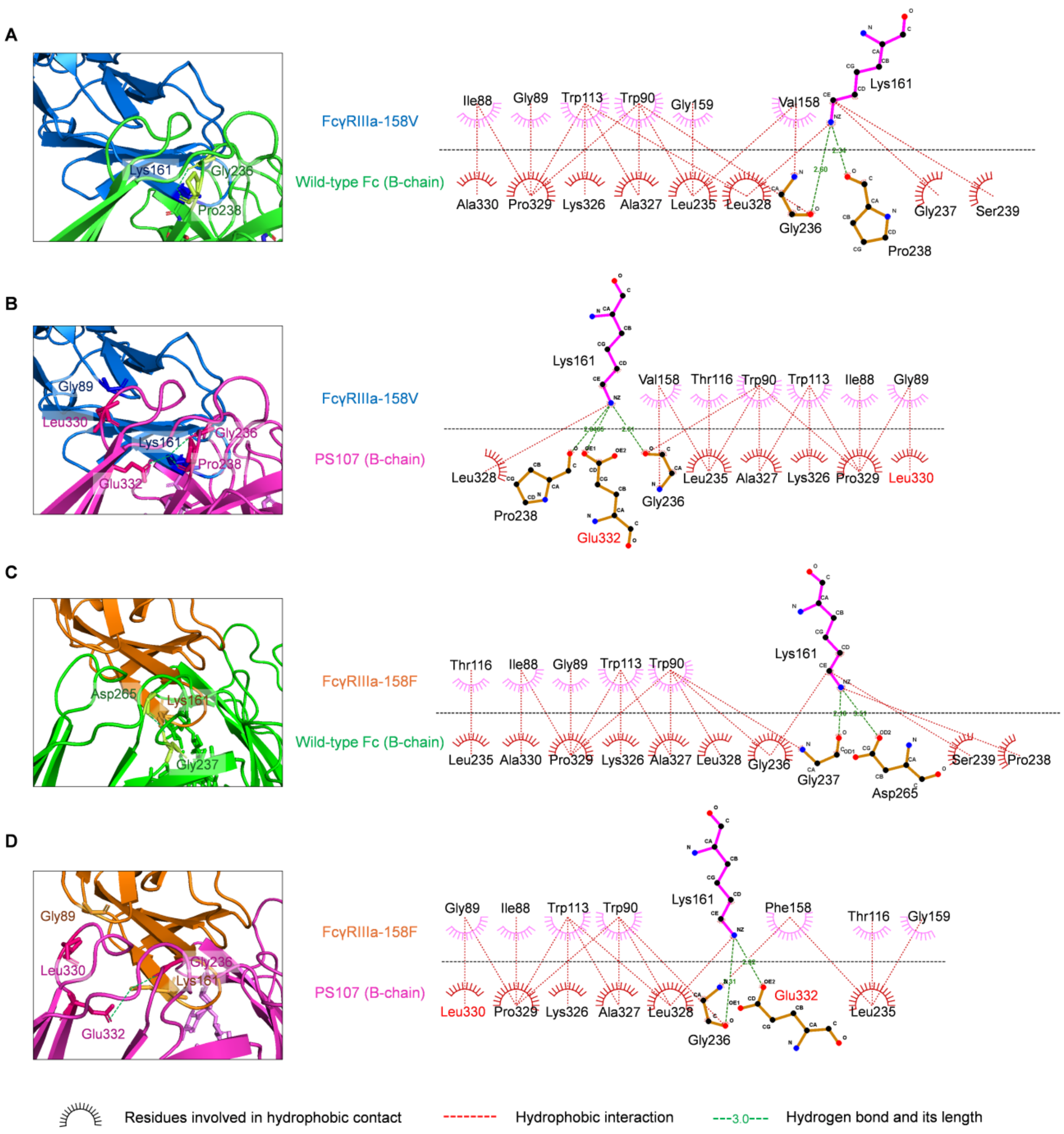
A
Lys161
Gly236
Pro238
FcγRIIIa-158V
Wild-type Fc (B-chain)
Ile88
Gly89
Trp113
Trp90
Gly159
Val158
Lys161
Ala330
Pro329
Lys326
Ala327
Leu235
Leu328
Gly236
Gly237
Ser239
Pro238
B
Gly89
Leu330
Lys161
Gly236
Pro238
Glu332
FcγRIIIa-158V
PS107 (B-chain)
Lys161
Val158
Thr116
Trp90
Trp113
Ile88
Gly89
Leu328
Leu235
Ala327
Lys326
Pro329
Leu330
Pro238
Gly236
Glu332
C
Asp265
Lys161
Gly237
FcγRIIIa-158F
Wild-type Fc (B-chain)
Thr116
Ile88
Gly89
Trp113
Trp90
Lys161
Leu235
Ala330
Pro329
Lys326
Ala327
Leu328
Gly236
Gly237
Asp265
Ser239
Pro238
D
Gly89
Leu330
Gly236
Lys161
Glu332
FcγRIIIa-158F
PS107 (B-chain)
Gly89
Ile88
Trp113
Trp90
Lys161
Phe158
Thr116
Gly159
Leu330
Pro329
Lys326
Ala327
Leu328
Glu332
Leu235
Gly236
Residues involved in hydrophobic contact
Hydrophobic interaction
---3.0---
Hydrogen bond and its length

Supplementary Fig. 23 In silico immunogenicity analysis of Fc variants. Heatmaps depicting predicted MHC class II binding for wild-type Fc and Fc variants (VLPLL, DE, PS101, PS102, and PS107). The x-axis represents overlapping 15-mer peptides derived from each Fc sequence, and the y-axis indicates the 27 most frequently occurring HLA class II alleles. Each value reflects the percentile rank of the predicted binding affinity, with lower ranks indicating higher predicted affinity.

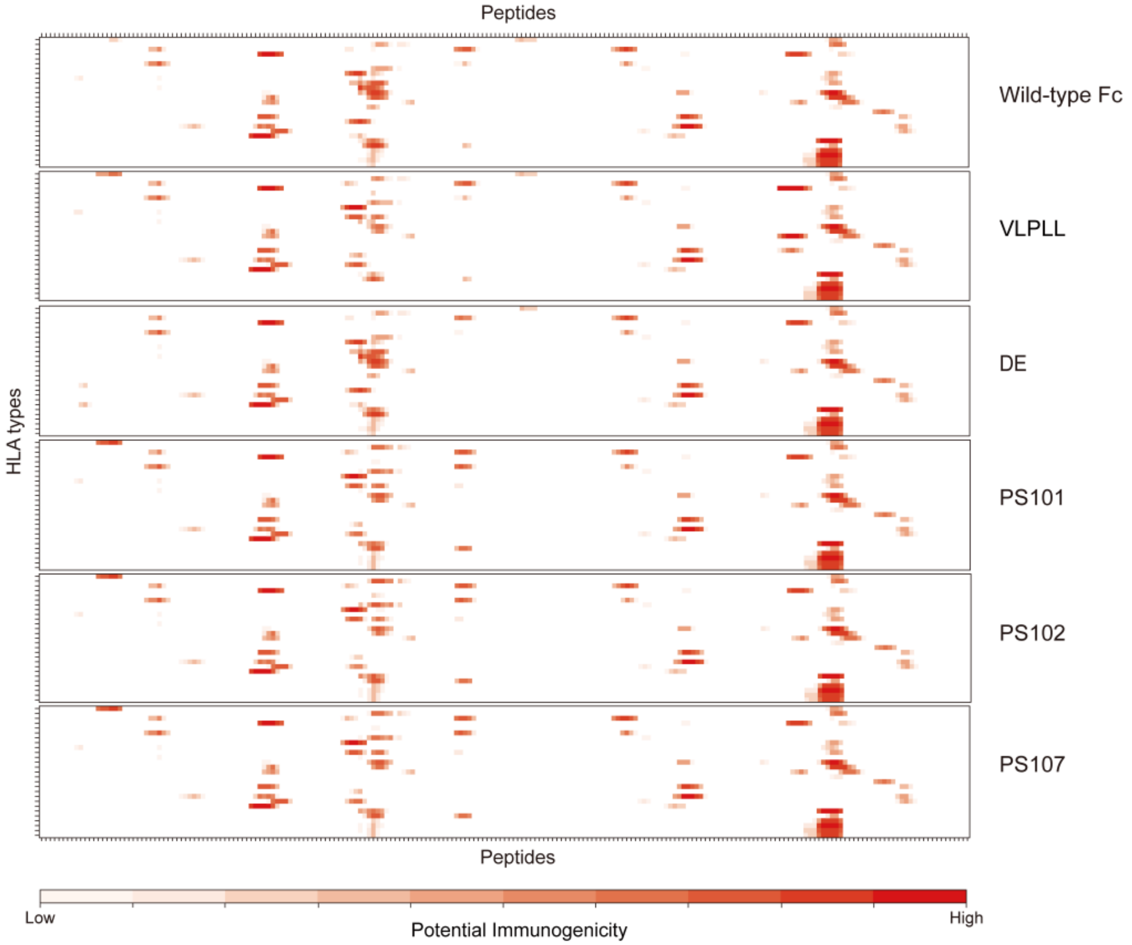

Supplementary Tables

Supplementary Table 1. Plasmids used in this study.

Plasmid name	Relevant characteristics	Reference
pcDNA5-FRT	CMV promoter, flp recombination target (FRT) site, hygromycin-B resistance gene encoded	Invitrogen (V601020)
pcDNA5-Igκ-Fc-FLAG-PDGFR	*Igκ-Fc-FLAG-PDGFR* gene in pcDNA5-FRT	This study
pcDNA5-Igκ-Fc-TL-FLAG-PDGFR	*Igκ-Fc(T299L)-FLAG-PDGFR* gene in pcDNA5-FRT	This study
pOG44-Flp	CMV promoter, temperature-sensitive Flp recombinase (flp-F70L) gene encoded	Invitrogen (V600520)
pMAZ-FcγRIIIa-158V-FLAG-Streptavidin-His	*FcγRIIIa$_{158V}$-FLAG-streptavidin* gene in pMAZ-IgH-GlycoT	[4]
pMAZ-FcγRIIb-FLAG-Streptavidin-His	*FcγRIIb-FLAG-streptavidin* gene in pMAZ-IgH-GlycoT	This study
pcDNA5-Igκ-Fc-PS101-FLAG-PDGFR	*Igκ-Fc-PS101-FLAG-PDGFR* gene in pcDNA5-FRT	This study
pcDNA5-Igκ-Fc-PS102-FLAG-PDGFR	*Igκ-Fc-PS102-FLAG-PDGFR* gene in pcDNA5-FRT	This study
pcDNA5-Igκ-Fc-PS107-FLAG-PDGFR	*Igκ-Fc-PS107-FLAG-PDGFR* gene in pcDNA5-FRT	This study
pMAZ-IgH-GlycoT	*Trastuzumab H chain* gene in pMAZ-IgH-H23	[1]
pMAZ-IgL-GlycoT	*Trastuzumab L chain* gene in pMAZ-IgL-H23	[1]
pMAZ-IgH-trastuzumab-VLPLL	*Trastuzumab VLPLL mutant H chain* gene in pMAZ-IgL	This study
pMAZ-IgH-trastuzumab-DE	*Trastuzumab DE mutant H chain* gene in pMAZ-IgL	This study
pMAZ-IgH-trastuzumab-PS101	*Trastuzumab PS101 mutant H chain* gene in pMAZ-IgL	This study
pMAZ-IgH-trastuzumab-PS102	*Trastuzumab PS102 mutant H chain* gene in pMAZ-IgL	This study
pMAZ-IgH-trastuzumab-PS107	*Trastuzumab PS107 mutant H chain* gene in pMAZ-IgL	This study
pMAZ-FcγRI-GST	*FcγRI-GST* gene in pMAZ-IgH-GlycoT	This study

pMAZ-FcγRIIa-131H-GST	*FcγRIIa$_{131H}$-GST* gene in pMAZ-IgH-GlycoT	[3]
pMAZ-FcγRIIa-131R-GST	*FcγRIIa$_{131R}$-GST* gene in pMAZ-IgH-GlycoT	[3]
pMAZ-FcγRIIIa-158V-GST	*FcγRIIIa$_{158V}$-GST* gene in pMAZ-IgH-GlycoT	[3]
pMAZ-FcγRIIIa-158F-GST	*FcγRIIIa$_{158F}$-GST* gene in pMAZ-IgH-GlycoT	[3]
pMAZ-FcγRIIb-GST	*FcγRIIb-GST* gene in pMAZ-IgH-GlycoT	[4]
pcDNA3-FcRn-GST	Amp^r CMV promoter, *FcRn-GST* gene in pcDNA3	[8]
pMAZ-FcγRIIIa-158V-His	*Monomeric FcγRIIIa-158V* gene with 6× His tag in pMAZ-IgL-GlycoT	[6]
pMAZ-FcγRIIIa-158F-His	*Monomeric FcγRIIIa-158F* gene with 6× His tag in pMAZ-IgL-GlycoT	This study
pMAZ-FcγRIIb-His	*Monomeric FcγRIIb* gene with 6× His tag in pMAZ-IgL-GlycoT	This study
pMAZ-HER2-His	*HER2-His* gene in pMAZ-IgL	[9]
pcDNA5-hFcγRIIIa-158V	*Human FcγRIIIa-158V* gene in pcDNA5-FRT	This study
pcDNA5-hFcγRIIIa-158F	*Human FcγRIIIa-158F* gene in pcDNA5-FRT	This study
pcDNA5-cFcγRIII	*Cynomolgus monkey FcγRIII* gene in pcDNA5-FRT	This study
pMAZ-IgL-rituximab	*Rituximab L chain* gene in pMAZ-IgL	[2]
pMAZ-IgH-rituximab	*Rituximab H chain* gene in pMAZ-IgL	[2]
pMAZ-IgH-rituximab-VLPLL	*Rituximab VLPLL mutant H chain* gene in pMAZ-IgL	This study
pMAZ-IgH-rituximab-DE	*Rituximab DE mutant H chain* gene in pMAZ-IgL	This study
pMAZ-IgH-rituximab-PS101	*Rituximab PS101 mutant H chain* gene in pMAZ-IgL	This study
pMAZ-IgH-rituximab-PS102	*Rituximab PS102 mutant H chain* gene in pMAZ-IgL	This study
pMAZ-IgH-rituximab-PS107	*Rituximab PS107 mutant H chain* gene in pMAZ-IgL	This study
pMAZ-IgL-cetuximab	*Cetuximab L chain* gene in pMAZ-IgL	This study
pMAZ-IgH-cetuximab	*Cetuximab H chain* gene in pMAZ-IgL	This study

pMAZ-IgH-cetuximab-VLPLL	*Cetuximab VLPLL mutant H chain* gene in pMAZ-IgL	This study
pMAZ-IgH-cetuximab-DE	*Cetuximab DE mutant H chain* gene in pMAZ-IgL	This study
pMAZ-IgH-cetuximab-PS101	*Cetuximab PS101 mutant H chain* gene in pMAZ-IgL	This study
pMAZ-IgH-cetuximab-PS102	*Cetuximab PS102 mutant H chain* gene in pMAZ-IgL	This study
pMAZ-IgH-cetuximab-PS107	*Cetuximab PS107 mutant H chain* gene in pMAZ-IgL	This study
pMAZ-mFcγRI-GST	*Mouse FcγRI-GST* gene (uniport code: P26151) in pMAZ-IgL	This study
pMAZ-mFcγRIIb-GST	*Mouse FcγRIIb-GST* gene (uniport code: P08101) in pMAZ-IgL	This study
pMAZ-mFcγRIII-GST	*Mouse FcγRIII-GST* gene (uniport code: P08508) in pMAZ-IgH-GlycoT	This study
pMAZ-mFcγRIV-GST	*Mouse FcγRIV-GST* gene (uniport code: A0A0B4J1G0) in pMAZ-IgL	This study
pMAZ-mFcRn α chain-GST	*Mouse FcRn alpha chain-GST* gene (uniport code: Q6PKB0) in pMAZ-IgL	This study
pMAZ-mβ2m-GST	*Mouse beta 2 microglobulin-GST* gene (uniport code: P01887) in pMAZ-IgL	This study
pMAZ-cFcγRI-GST	*Cynomolgus monkey FcγRI-GST* gene (uniport code: Q8SPW5) in pMAZ-IgL	This study
pMAZ-cFcγRIIa-GST	*Cynomolgus monkey FcγRIIa-GST* gene (uniport code: Q8SPW4) in pMAZ-IgL	This study
pMAZ-cFcγRIIb-GST	*Cynomolgus monkey FcγRIIb-GST* gene (uniport code: Q8SPW3) in pMAZ-IgL	This study
pMAZ-cFcγRIII-GST	*Cynomolgus monkey FcγRIII-GST* gene (uniport code: Q8SPW2) in pMAZ-IgL	This study
pMAZ-cFcRn α chain-GST	*Cynomolgus monkey FcRn alpha chain-GST* gene (uniport code: Q8SPV9) in pMAZ-IgL	This study
pMAZ-cβ2m-GST	*Cynomolgus monkey beta 2 microglobulin-GST* gene (uniport code: I7GKX8) in pMAZ-IgL	This study

Supplementary Table 2. Oligonucleotide primers used in this study.

Primer name	Oligonucleotide sequence (5'→3')
MJ#296	CGCAGCGAGCTAGCATGGAGACAGACACACTCCTGCTATGGGTACTGCTG CTCTGGGTTCCAGGTTCCACTGGTGAC GG GGCCCAGCCGGCC
MJ#309	GGCGCGCCCTTTGTCATCGTCATCTTTATAATCGGCCCCCGAGGCCCC
MJ#256	GCTGTGGGCCAGGACACGCAGGAGG
MJ#257	ATCACCACCACCTTAAAGGGCAAGGAGTGTGGCACCACGATGACCTCCTG CGTGTCCTGG
MJ#258	CCCTTTAAGGTGGTGGTGATCTCAGCCATCCTGGCCCTGGTGGTGCTCACC ATCATCTCC
MJ#259	ACGTGGCTTCTTCTGCCAAAGCATGATGAGGATGATAAGGGAGATGATGG TGAGCACC
MJ#310	GATTATAAAGATGACGATGACAAAGGGCGCGCCGCTGTGGGCCAGGACA CG
MJ#272	TTAGGGAAGCTTTCAACGTGGCTTCTTCTGCCAAAGC
MJ#267	GACAAGAAAGTTGAGCCCCCTAAATCTTGTGACAAAACTCACACATGCCCA CCG
MJ#50	CCCTAAAATCTAGATCATTTACCCGGGGACAGGGAGAGG
MJ#616	GACAAGAAAGTTGAGCCCCCTAAATCTTGTGACAAAACTCGCACATGCCCA CCG
MJ#49	CGCAGCGAGCGCGCACTCCGAGGTCCAACTGGTCGAAAGCG
MJ#268	ACAAGATTTAGGGGGCTCAACTTTCTTGTC
MJ#545	CGCAGCGAGCGCGCACTCCCAGGTCCAGCTCCAACAGCC
MJ#546	ACAAGATTTGGGCTCTGCTTTCTTGTC
MJ#547	GACAAGAAAGCAGAGCCCAAATCTTGTGACAAAACTCACACATGCCCACC G
MJ#548	GACAAGAAAGCAGAGCCCAAATCTTGTGACAAAACTCGCACATGCCCACC G
MJ#549	CGCAGCGAGCGCGCACTCCCAGGTGCAGCTGAAGCAGAGC
MJ#550	ACAAGATTTGGGCTCAACTTTCTTGTCC
MJ#551	GGACAAGAAAGTTGAGCCCAAATCTTGTGACAAAACTCACACATGCCCACC G
MJ#552	GGACAAGAAAGTTGAGCCCAAATCTTGTGACAAAACTCGCACATGCCCAC CG
MJ#573	CGCAGCGAGCGCGCACTCC GGCATGCGGACTGAAGATCTCC
MJ#574	CCCTAAAATCTAGATTATCAATGATGATGGTGGTGATGGCTGCCTTGGTAC CCAGGTGGAAAGAATGATGAG
MJ#575	CGCAGCGAGCGCGCACTCCGCTCCCCCAAAGGCTGTGC
MJ#576	CCCTAAAATCTAGATTATCAATGATGATGGTGGTGATGGCTGCCGGGAGCT TGGACAGTGATGGTCAC

MJ#565	CGCAGCGAGCTAGCATGGAGATGTGGCAGCTGCTGCTGCCTACAGCTCTG CTGCTGCTGGTGAGTGCT GGCATGCGGACTGAAGATCTCC
MJ#566	CAAACAGCAGCACCATCACCAGGCAGAAGCTCACTTGGTACCCAGGTGGA AAGAATGATGAG
MJ#567	TTAGGGAAGCTTTCACACGCTGAAGTACAGGCCGGTGTCCACGGCAAACA GCAGCACCATCACCAGG
MJ#602	CGCAGCGAGCTAGCATGGAGATGTGGCAGCTGCTGCTGCCTACAGCTCTG CTGCTGCTGGTGAGTGCT GGGATGAGAGCTGAGGATCTGCCC
MJ#603	CAAACAGCAGCACCATCACCAGGCAGAAGCTCACCTGATAGCCAGGGGGG AAGAAGC
MJ#604	TTAGGGAAGCTTTCACATGCTGAAGTACAGGCCGGTGTCCACGGCAAACA GCAGCACCATCACCAGG
MJ#273	CGCAGCGAGGCCCAGCCGGCCGACAAAACTCACACATGCCCACCG
MJ#418	CCGGGAGATCATGAGGGTGTCCTTGGGTTTTGGGGGRARGAGGAAGACT GA CGGTCCCCCCAGGAGTTCAG
MJ#419	CCGGGAGATCATGAGGGTGTCCTTGGGTTTTGGGGGRARGAGGAAGACG TC CGGTCCCCCCAGGAGTTCAG
MJ#420	CCGGGAGATCATGAGGGTGTCCTTGATTTTTGGGGGRARGAGGAAGACTG A CGGTCCCCCCAGGAGTTCAG
MJ#421	CCGGGAGATCATGAGGGTGTCCTTGATTTTTGGGGGRARGAGGAAGACGT C CGGTCCCCCCAGGAGTTCAG
MJ#422	AAGGACACCCTCATGATCTCCCGG
MJ#423	CTCCTTGCCATTCAGCCAGTCCTGGTGCAGGACGGTGAGRAYGCTGACCAC ACGCAGCGTGCTGTTGTACTGCTCCTCRSGCGGCTTTGTCTTGGCATTATGC ACC
MJ#424	CTCCTTGCCATTCAGCCAGTCCTGGTGCAGGACGGTGAGRAYGCTGACCAC ACGGTACGTGCTGTTGTACTGCTCCTCRSGCGGCTTTGTCTTGGCATTATGC ACC
MJ#425	CAGGACTGGCTGAATGGCAAGGAGTACAAGTGCAAGGTCTCCAACAAAGC CCTCCCAGCCCCCATCGAGAAAACCATCTCCAAAGCCAAAGGGCAGCCCCG AGAACC
MJ#426	CAGGACTGGCTGAATGGCAAGGAGTACAAGTGCAAGGTCTCCAACAAAGC CCTCCCAGCCCCCATCGAGAAAACCATCTCCAAACAGAAAGGGCAGCCCC GAGAACC
MJ#427	CAGGACTGGCTGAATGGCAAGGAGTACAAGTGCAAGGTCTCCAACAAAGC CCTCCCAGCCCCCGAGGAGAAAACCATCTCCAAAGCCAAAGGGCAGCCCC GAGAACC
MJ#428	CAGGACTGGCTGAATGGCAAGGAGTACAAGTGCAAGGTCTCCAACAAAGC CCTCCCAGCCCCCGAGGAGAAAACCATCTCCAAACAGAAAGGGCAGCCCC GAGAACC

MJ#429	CAGGACTGGCTGAATGGCAAGGAGTACAAGTGCAAGGTCTCCAACAAAGCCCTCCCACTGCCCATCGAGAAAACCATCTCCAAAGCCAAAGGGCAGCCCCGAGAACC
MJ#430	CAGGACTGGCTGAATGGCAAGGAGTACAAGTGCAAGGTCTCCAACAAAGCCCTCCCACTGCCCATCGAGAAAACCATCTCCAAACAGAAAGGGCAGCCCCGAGAACC
MJ#431	CAGGACTGGCTGAATGGCAAGGAGTACAAGTGCAAGGTCTCCAACAAAGCCCTCCCACTGCCCGAGGAGAAAACCATCTCCAAAGCCAAAGGGCAGCCCCGAGAACC
MJ#432	CAGGACTGGCTGAATGGCAAGGAGTACAAGTGCAAGGTCTCCAACAAAGCCCTCCCACTGCCCGAGGAGAAAACCATCTCCAAACAGAAAGGGCAGCCCCGAGAACC
MJ#433	GCCGTCGGAGTCCAGCACCAGAGGCGTGGTCTTGTAGTTGTTCTCC
MJ#434	GCCGTCGGAGTCCAGCACGGGAGGCGTGGTCTTGTAGTTGTTCTCC
MJ#435	GTGCTGGACTCCGACGGC
MJ#274	CTTAACGCGGCCCCCGAGGCCCC TTTACCCGGGGACAGGGAGAGG
MJ#629	GATATAATACCATATATTGCCTATAAGAGAATGCTCAC
MJ#630	GCTCCAAGCTCTGGCCCCTAC
MJ#625	GGTCACATATTTACAGAATGGCAAAGG
MJ#626	CAGTCTCTGAAGACACATTTTTACTCCCAAC
MJ#627	TCCTACTTCTGCAGGGGGCTTT
MJ#628	GGGACTTTTGGGGACCTCCTG

Supplementary Table 3. Analysis of pharmacokinetic parameters in hFcRn Tg mice.

		Trastuzumab	Trastuzumab-VLPLL	Trastuzumab-DE	Trastuzumab-PS101	Trastuzumab-PS102
$t_{1/2}$	(h)	332.72 ± 28.96	239.52 ± 9.08	138.03 ± 5.03	128.60 ± 1.01	120.21 ± 2.28
AUC_{last}	(μg/ml×h)	23349.22 ± 3479.90	19191.00 ± 672.72	12106.86 ± 288.27	5071.74 ± 328.55	6219.26 ± 80.52
AUC_{inf}	(μg/ml×h)	24771.24 ± 3969.81	19418.63 ± 724.96	12123.91 ± 284.95	5081.15 ± 330.33	6226.31 ± 80.23
$AUC_{\%}$		5.54 ± 1.76	1.16 ± 0.23	0.14 ± 0.03	0.18 ± 0.02	0.11 ± 0.01

Supplementary Table 4. Analysis of pharmacokinetic parameters in NSG hFcRn Tg mice,

		Trastuzumab			Trastuzumab-DE			Trastuzumab-PS101			Trastuzumab-PS102		
$t_{1/2}$	(h)	112.74	±	47.48	65.15	±	1.31	70.43	±	3.47	76.93	±	14.99
AUC_{last}	(μg/ml×h)	8595.12	±	2678.56	7640.69	±	83.88	8932.64	±	216.05	5766.71	±	1083.64
AUC_{inf}	(μg/ml×h)	9759.94	±	1830.6 3	7641.67	±	84.09	8933.64	±	216.42	5767.86	±	1084.21
$AUC_{\%}$		0.86	±	0.11	1.00	±	0.00	1.00	±	0.00	1.00	±	0.00

References

1. Jung ST, Reddy ST, Kang TH, Borrok MJ, Sandlie I, Tucker PW, et al. Aglycosylated IgG variants expressed in bacteria that selectively bind FcγRI potentiate tumor cell killing by monocyte-dendritic cells. Proc Natl Acad Sci. 2010;107(2):604-9.

2. Ko S, Park S, Sohn MH, Jo M, Ko BJ, Na JH, et al. An Fc variant with two mutations confers prolonged serum half-life and enhanced effector functions on IgG antibodies. Exp Mol Med. 2022;54(11):1850-61.

3. Jung ST, Kelton W, Kang TH, Ng DT, Andersen JT, Sandlie I, et al. Effective phagocytosis of low Her2 tumor cell lines with engineered, aglycosylated IgG displaying high FcγRIIa affinity and selectivity. ACS Chem Biol. 2013;8(2):368-75.

4. Jo M, Kwon HS, Lee KH, Lee JC, Jung ST. Engineered aglycosylated full-length IgG Fc variants exhibiting improved FcγRIIIa binding and tumor cell clearance. mAbs. 2018;10(2):278-89.

5. Kawarasaki Y, Griswold KE, Stevenson JD, Selzer T, Benkovic SJ, Iverson BL, et al. Enhanced crossover SCRATCHY: construction and high-throughput screening of a combinatorial library containing multiple non-homologous crossovers. Nucleic Acids Res. 2003;31(21):e126.

6. Yoon HW, Jo M, Ko S, Kwon HS, Lim CS, Ko BJ, et al. Optimal combination of beneficial mutations for improved ADCC effector function of aglycosylated antibodies. Mol Immunol. 2019;114:62-71.

7. Vilches C, Castaño J, Muñoz P, Peñalver J. Simple genotyping of functional polymorphisms of the human immunoglobulin G receptors CD16A and CD32A: a reference cell panel. Tissue Antigens. 2008;71(3):242-6.

8. Berntzen G, Lunde E, Flobakk M, Andersen JT, Lauvrak V, Sandlie I. Prolonged and increased expression of soluble Fc receptors, IgG and a TCR-Ig fusion protein by transiently transfected adherent 293E cells. J Immunol methods. 2005;298(1-2):93-104.

9. Ko S, Jo M, Kyung M, Lee W, Ko WH, Na J-H, et al. Engineering FcRn binding kinetics dramatically extends antibody serum half-life and enhances therapeutic potential. J Biol Eng. 2025;19(1):35.
